# Integrative genomics analyses unveil downstream biological effectors of disease-specific polymorphisms buried in intergenic regions

**DOI:** 10.1038/npjgenmed.2016.6

**Published:** 2016-04-27

**Authors:** Haiquan Li, Ikbel Achour, Lisa Bastarache, Joanne Berghout, Vincent Gardeux, Jianrong Li, Younghee Lee, Lorenzo Pesce, Xinan Yang, Kenneth S Ramos, Ian Foster, Joshua C Denny, Jason H Moore, Yves A Lussier

**Affiliations:** 1 BIO5 institute, University of Arizona, Tucson, AZ, USA; 2 Department of Medicine, University of Arizona, Tucson, AZ, USA; 3 Department of Medicine, University of Illinois at Chicago, IL, USA; 4 Section of Genetic Medicine, Department of Medicine, University of Chicago, Chicago, IL, USA; 5 Center for Biomedical Informatics, Department of Medicine, University of Chicago, Chicago, IL, USA; 6 Departments of Biomedical Informatics and Medicine, Vanderbilt University, TN, USA; 7 Computation Institute, Argonne National Laboratory and University of Chicago, IL, USA; 8 Department of Pediatrics, University of Chicago, Chicago, IL, USA; 9 Mathematics and Computer Science Division, Argonne National Laboratory, Chicago, IL, USA; 10 Department of Computer Science, University of Chicago, Chicago, IL, USA; 11 Department of Genetics, Institute for Quantitative Biomedical Sciences, Geisel School of Medicine, Dartmouth College, Hanover, NH, USA; 12 Penn Institute for Biomedical Informatics & Department of Biostatistics and Epidemiology & Department of Genetics, Perelman School of Medicine, The University of Pennsylvania, PA, USA; 13 Institute for Genomics and Systems Biology, Argonne National Laboratory & University of Chicago, IL, USA; 14 University of Arizona Cancer Center, University of Arizona, Tucson, AZ, USA; 15 Section for Bioinformatics, Department of Bioengineering, University of Illinois at Chicago, IL, USA; 16 Department of Biopharmaceutical Sciences, University of Illinois at Chicago, IL, USA

## Abstract

Functionally altered biological mechanisms arising from disease-associated polymorphisms, remain difficult to characterise when those variants are intergenic, or, fall between genes. We sought to identify shared downstream mechanisms by which inter- and intragenic single-nucleotide polymorphisms (SNPs) contribute to a specific physiopathology. Using computational modelling of 2 million pairs of disease-associated SNPs drawn from genome-wide association studies (GWAS), integrated with expression Quantitative Trait Loci (eQTL) and Gene Ontology functional annotations, we predicted 3,870 inter–intra and inter–intra SNP pairs with convergent biological mechanisms (FDR<0.05). These prioritised SNP pairs with overlapping messenger RNA targets or similar functional annotations were more likely to be associated with the same disease than unrelated pathologies (OR>12). We additionally confirmed synergistic and antagonistic genetic interactions for a subset of prioritised SNP pairs in independent studies of Alzheimer’s disease (entropy *P*=0.046), bladder cancer (entropy *P*=0.039), and rheumatoid arthritis (PheWAS case–control *P*<10^−4^). Using ENCODE data sets, we further statistically validated that the biological mechanisms shared within prioritised SNP pairs are frequently governed by matching transcription factor binding sites and long-range chromatin interactions. These results provide a ‘roadmap’ of disease mechanisms emerging from GWAS and further identify candidate therapeutic targets among downstream effectors of intergenic SNPs.

## Introduction

The abundance of newly discovered disease-associated polymorphisms now enables inquiries about their summative and interactive effects.^1^ Since 2005, genome-wide association studies (GWAS) have reported >15,000 single-nucleotide polymorphisms (SNPs) associated with over 1,200 complex diseases and traits.^2^ From these studies, we have learned that half of the disease-associated SNPs reside within poorly characterised intergenic regions. Although downstream effects of missense and nonsense coding SNPs can be investigated straightforwardly in cellular and animal models, effects arising from intergenic SNPs remain largely uncharacterised and are often challenging to validate experimentally using *in vitro* and *in vivo* assays.

Computational biology can potentially bridge the mechanistic gap between detecting disease-associated SNPs and providing biological interpretations of how different risk loci contribute to disease incidence and prevalence. We and others have shown that systematically integrating studies of protein–protein interaction with experimentally verified disease-associated coding SNPs enables discovery of new disease-gene candidates and testable associations between biological pathways and disease.^3–7^ Other disease-mechanism-based methods have prioritised GWAS signals by leveraging prior biological knowledge inferred from the physical proximity of SNPs to gene loci^8–11^ or from expression quantitative loci (eQTL) associations.^12–17^ Recent high-throughput genomics projects such as The Encyclopedia of DNA Elements (ENCODE) have extended quantitative measures of biological activity into intergenic regions.^18,19^ These projects led to integrative genomic analyses and systemic mapping of disease-associated SNPs to regulatory elements, including enhancers, transcription factor (TF) binding sites or chromatin accessibility marks.^20–25^ Nonetheless, analysis of how downstream disease mechanisms emerge from intergenic SNPs located in biologically active regulatory genomic regions remains elusive.

We hypothesised that the mechanisms by which polymorphisms contribute to disease risk can be unveiled by systematically analysing their downstream transcriptomic effects. The functional convergence of intergenic SNPs with intragenic ones may influence the course of disease via the same mechanisms. Building on eQTL and ENCODE data, we approached this hypothesis by identifying shared molecular and biological mechanisms by which two SNPs (irrespective of their genomic location but not in linkage disequilibrium) are associated with the same disease. We developed a computational method focused on ascertaining and quantifying disease mechanisms of SNPs with known disease relationships from the National Human Genome Research Institute (NHGRI) GWAS catalogue (e.g., Lead SNPs), which are also associated with altered messenger RNA (mRNA) expression levels via eQTL studies. We first devised a systematic method to identify overlap and similarity of biological activities shared between every two SNPs (e.g., mRNA expression, inferred molecular function and biological processes). Second, in support of the predicted shared mechanisms between SNPs associated with the same disease, we provided additional independent evidence by: (i) exploring non-additive synergetic and antagonistic SNP–SNP interactions in GWAS of bladder cancer, Alzheimer’s disease and rheumatoid arthritis (RA), and (ii) utilising ENCODE-derived data to identify Lead SNP pairs located in similar regulatory regions that might explain their shared downstream biological mechanisms. We focused our investigation on Lead SNP pairs comprised of at least one intergenic SNP.

## Results

### Approach overview

**Figure 1:**
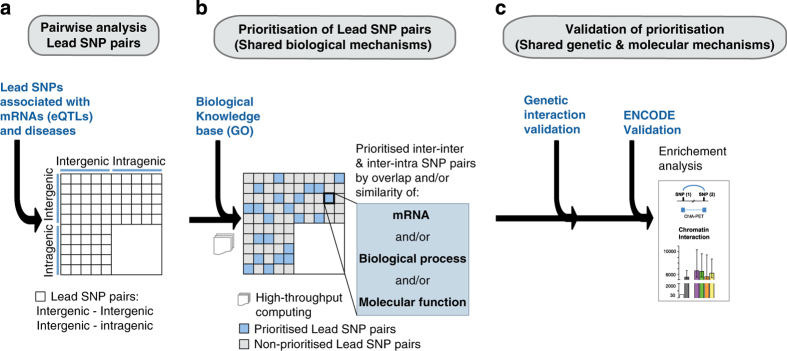
Schematic of Lead SNP pair prioritisation methods. (**a**) Lead SNP pairs analysed in this study contain at least one intergenic SNP and are associated with one or more of 467 diseases in the NHGRI GWAS catalogue and with gene expression levels (6,301 mRNAs) derived from a lymphoblastoid cell line eQTL study. Although computed, pairs consisting of two intragenic SNPs were not the main focus of this study (blank in matrix). (**b**) Lead SNP pairs were prioritised and controlled with empirical scale-free networks to yield significant Lead SNP pairs sharing at least one of the three imputed biological mechanisms (blue highlighted squares). Biological knowledge bases refer to eQTL associations and gene ontology annotations of molecular functions and biological process. (**c**) Prioritised inter–inter and inter–intra Lead SNP pairs were further validated for genetic interaction using three independent association studies (GWAS and PheWAS), and for shared TFs and interacting regulatory elements using ENCODE-derived data sets.

To determine intergenic SNPs’ contribution to disease risk, we computationally imputed biological mechanisms that are common to more than one intergenic Lead SNP associated with the same disease. We analysed Lead SNPs associated with any of the 467 diseases in the NHGRI GWAS catalogue^2^ that had at least one eQTL association in the SCAN database,^26^ derived from lymphoblastoid cell lines. This yielded 2,358 Lead SNPs (Supplementary Data S1; 1,092 intergenic) and each was paired across all possible combinations. Any pairs of SNPs that were in linkage disequilibrium with each other at *r*^2^⩾0.8 using HapMap data for the CEU population were removed from our analysis (see details in Materials and Methods section). Lead SNP pairs were categorised into three groups based on assertions by dbSNP (Build 138):^27^ intergenic–intergenic (inter–inter) pairs when both SNPs are at least 2,000 bp 5ʹ and 500 bp 3ʹ of protein-coding gene coordinates, intergenic–intragenic (inter–intra) pairs when one SNP is intergenic and the other is within gene coordinates, and intragenic–intragenic (intra-intra) pairs in cases where both SNPs were found within or near gene coordinates. This study focused on pairs of Lead SNPs comprised of at least one intergenic SNP (inter–inter or inter–intra), which left two million pairwise combinations (Figure 1a and Supplementary Figure S1a,b). For each Lead SNP, we determined the mRNA transcripts that were associated by eQTL (median 2 transcripts per SNP) and retrieved their biological processes (GO–BP) and molecular function (GO–MF) annotations from the Gene Ontology (GO 5/19/2009^28^). These annotations allowed us to prioritise SNP pairs (inter–inter and inter–intra) on the basis of having the same or similar functional biological mechanisms, even when the exact mRNA target is distinct (e.g., receptor-ligand, signalling pathway and protein complexes). These data were then overlapped between each SNP comprising an inter–inter or inter–intra Lead SNP pair.^28,29^

To evaluate the significance of imputed biological mechanisms, we developed stringent prioritisation methods by mRNA overlap, GO–MF similarity and GO–BP similarity controlled empirically with scale-free networks^3,30^ and applied these systematically to the two million surveyed Lead SNP pairs. Pairs exhibiting sufficient overlap and/or similarity at FDR<0.05 were termed ‘prioritised Lead SNP pairs’ (Figure 1b and Supplementary Figure S1c). Computationally intensive empirical calculations were required owing to random distributions being anticonservative. We then performed enrichment analyses to assess whether shared biological mechanisms are more likely to be found among Lead SNP pairs related to the same disease rather than across distinct diseases. Leveraging ENCODE data, we evaluated shared regulatory properties and molecular mechanisms at play that relate prioritised Lead SNP pairs to the same disease. Finally, using genome-wide associations in independent data sets, we determined that prioritised Lead SNP pairs in rheumatoid arthritis, bladder cancer and Alzheimer's disease show non-additive synergetic genetic interactions, and that long-range interactions may explain converged biological effects of inter–inter and inter–intra Lead SNPs (Figure 1c and Supplementary Figure S1d).

### Substantial associations unveiled between Lead SNP pairs and biological mechanisms

**Figure 2:**
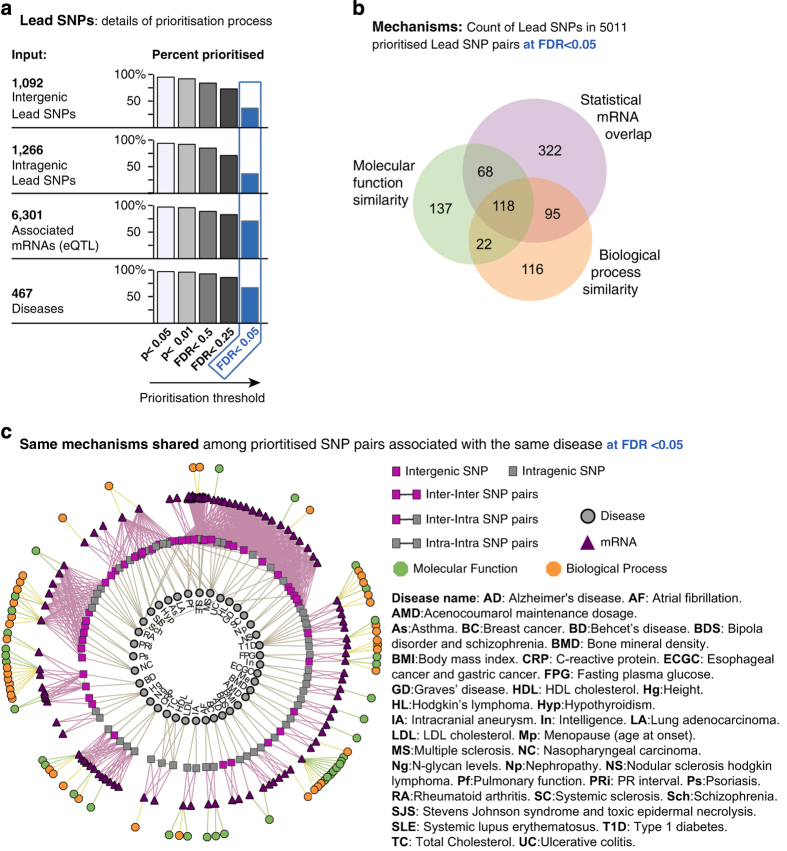
Surveyed Lead SNPs associated with mRNA expression and diseases found in prioritised Lead SNP pairs. Lead SNP pairs were prioritised by mRNAs overlap, molecular function similarity or biological process similarity (**a**) Input shown on the left, percentage of Lead SNPs in the prioritised SNP pairs and their associated mRNAs and diseases found among total surveyed Lead SNPs. Different *P* value and FDR cutoffs were applied to stratify SNP pair prioritisation and percent-derived Lead SNPs (bars). Results at FDR<0.05 (406 intergentic lead SNPs, 472 intragenic lead SNPs, 4,493 mRNA and 312 diseases; blue highlight) were selected for subsequent analyses. (**b**) Venn diagram of Lead SNPs in the 5,011 prioritised pairs according to mRNA overlap, molecular function similarity, and biological process similarity. (**c**) The network illustrates the subset of Lead SNP pairs where both SNPs had been associated with the same disease prioritised only by the overlap of mRNA, molecular function (GO–MF) or biological processes (GO–BP) at FDR<0.05, excluding GO terms found by similarity. Under this criterion, 72 (out of 105) prioritised Lead SNP pairs associated with the same disease. Five additional ones found by similarity of GO terms are not shown for visualisation clarity. 467 diseases were used as input (Materials and Methods).

We first applied the three prioritisation methods (statistical mRNA overlap, molecular function similarity and biological process similarity) separately to the two million surveyed Lead SNP pairs (2,358 SNPs) at False Discovery Rate (FDR)<0.05. This prioritised 5,011 total Lead SNP pairs, with 3,870 pairs containing at least one intergenic SNP (inter–inter and inter–intra pairs; Supplementary Table S1). In these 5,011 SNP pairs we observe 406 (37% of input) intergenic Lead SNPs and 472 (37%) intragenic Lead SNPs, with 4,493 (71%) associated mRNAs and corresponding to 312 (67%) diseases (Figure 2a). Details of the data distribution and composition can be found in Supplementary Data S1 and Supplementary Figure S2. One hundred eighteen SNPs appeared in a pair that was prioritised according to all three imputed mechanisms, with 303 Lead SNPs prioritised according to at least two imputed mechanisms and the remainder of 322 (mRNA overlap), 137 (molecular function similarity) and 116 (biological process similarity) Lead SNPs were reported in pairs exhibiting a single prioritisation mechanism (Figure 2b). To visualise shared mechanisms within a given disease, we selected prioritised SNP pairs (FDR<5%) where both SNPs had been identified by association to the same disease and illustrated common mRNA targets and overlapping GO annotations (Figure 2c). These results included 43 diseases, but for visual clarity five GWAS phenotypes (Crohn’s disease, immunoglobulin A levels, anorexia nervosa, prostate cancer and metabolic levels) which had highly similar but non-identical GO terms are not illustrated, although these are included in later analyses (Supplementary Data S3 and S4). These findings suggest that the three prioritisation methods were complementary and illustrate how genetic risk of disease arises, at least in part, from systems biology properties of shared mechanisms.

### Lead SNPs sharing biological mechanisms are enriched specifically within the same disease

To assess whether within-disease Lead SNPs were more likely to share biological mechanisms than SNPs associated with distinct diseases, we performed a set of enrichment analyses. Focusing on the 3,870 prioritised inter–inter and inter–intra Lead SNP pairs, we identified 80 pairs that relate to the same disease at FDR<0.05. Thirty-one SNPs were prioritised in two or more pairwise relationships for a total of 86 unique SNPs. Seven of these SNPs had exclusively *cis*-eQTL relationships, 44 had exclusively *trans*-eQTL relationships and 35 SNPs had both *cis* and *trans-*eQTLs.

**Figure 3:**
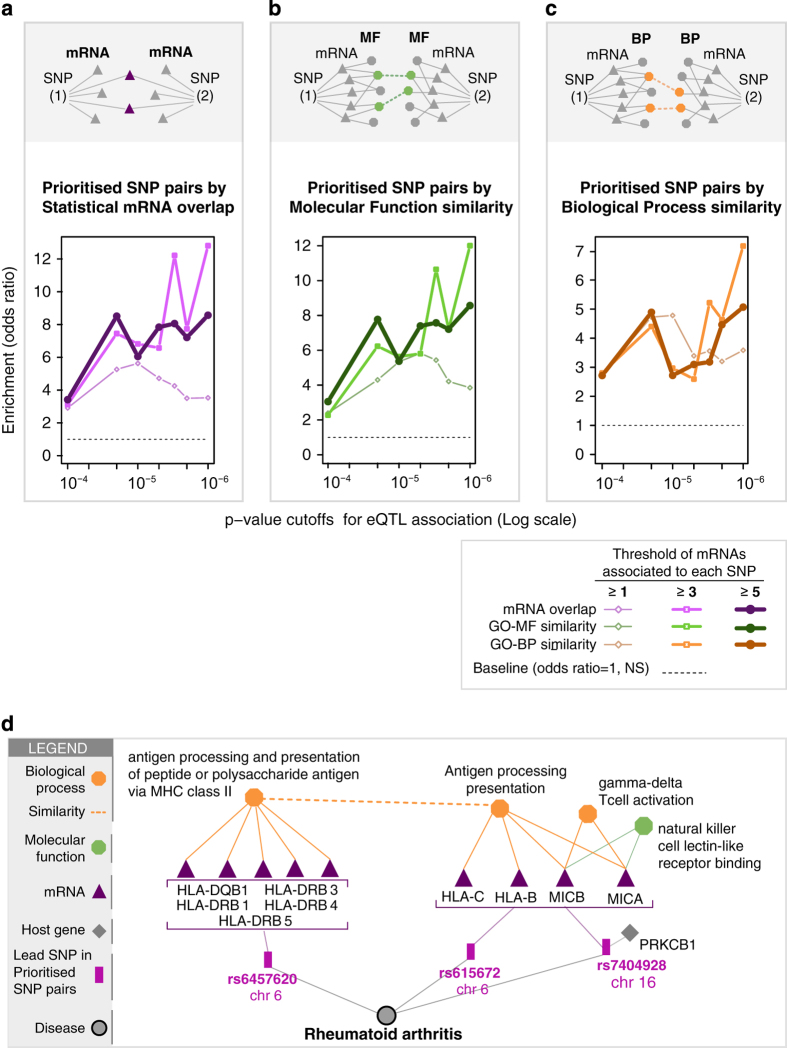
Enrichment of shared biological mechanisms among inter–inter and inter–intra Lead SNP pairs associated with the same disease. Three biological mechanisms were imputed for each Lead SNP-pair: mRNA overlap (**a**), similarity of molecular functions (**b**), and similarity of biological processes (**c**). Prioritised inter–inter and inter–intra Lead SNP pairs (100,000 permutation resampling; FDR<0.05) were significantly enriched when SNPs were prioritised in same disease versus across distinct diseases regardless of the *P* value cutoffs for eQTL association (*x* axis). ORs for inter–inter and inter–intra Lead SNP pairs ranged from 2.9 to 12.8 (1.3×10^−12^⩽*P*⩽6.5×10^−4^), 2.3 to 12.0 (3.3×10^−11^⩽*P*⩽6.5×10^−4^) and 2.6 to 7.2 (8.4×10^−17^⩽*P*⩽0.01) (**a**–**c**, respectively). (**d**) Subset of rheumatoid arthritis (RA) prioritised disease network. Among the 34 surveyed RA-associated Lead SNPs via GWAS and 138 mRNAs via eQTL (*P*<10^−4^), eight Lead SNPs were identified that related to 15 mRNAs, 14 GO–MF, and 23 GO–BP. Three of the eight Lead SNPs shared the same mRNAs and/or similar functional mechanisms. Full network appears in Supplementary Figure S7, only prioritised SNP pairs at FDR<0.05 and with significant SNP–GO–SNP associations (FDR<0.05) are shown. *r*^2^=0.004 between *rs6457620* and *rs615672* (HapMap Phase 3).

Twenty percent of the pairs (16/80) were comprised of SNPs mapping to two different chromosomes, whereas 64 pairs of SNPs were mapped to the same chromosome, although not within the same linkage disequilibrium (LD) block (Supplementary Figures S3 and S4). Involvement of HLA in prioritised diseases was prominent, with 11% (9/80) of SNP pairs including one marker that mapped within the HLA locus (Chr6: 29–34 Mb) with the other mapping to a different chromosome, 23% (18/80) of pairs were both outside of HLA and 67% (53/80) of pairs had both SNPs within HLA. The odds ratio (OR) in favour of Lead SNPs within the same disease sharing biological mechanisms is striking when compared SNP pairs where GWAS mapping was to two distinct diseases (one-sided Fisher’s Exact test; FET *P*=8.4×10^−17^; Figure 3). Specifically, when using the stringent *P* value cutoff of eQTL association (⩽3×10^−6^) and multiple mRNAs associated with each Lead SNP (threshold ⩾3), we observed substantial disease-specific enrichment with respect to mRNA overlap (OR=12, one-sided FET *P*=6.1×10^−9^; Figure 3a), GO–MF similarity (OR=11, one-sided FET *P*=3.9×10^−8^; Figure 3b), and GO–BP similarity (OR=5.2, one-sided FET *P*=2.3×10^−4^; Figure 3c). These results were also reproduced in a subset of inter–intra Lead SNP pairs (Supplementary Figure S5), or exclusively two intragenic SNPs (Supplementary Figure S6). Even in the absence of mRNA overlap from eQTL, Lead SNP pairs with similar biological functions between their respective mRNAs remain significantly enriched with disease-specific predictions (OR=3.9, one-sided FET *P*=6.8×10^−7^). As an example of functional convergence in prioritised SNP pairs that come from the same disease, we have illustrated the mRNA transcript overlap, molecular function similarity and biological process similarity observed for all SNP pairs associated with RA (Supplementary Figure S7). Among eight Lead SNPs associated with RA, *rs7404928, rs615672* and *rs6457620* were prioritised by eQTL to the same mRNA transcripts (as well as nonoverlapping mRNAs), and all prioritised SNPs converged towards immune response (GO:0006955) and/or antigen processing and presentation via MHC class I (GO:0002474) or class II (GO:0002586) through at least one path—including SNPs that mapped outside of the MHC region. This is consistent with what is known about the biology of RA, and the importance of antigen responses in pathology.^31^

We further confirmed the robustness of the disease-specific enrichment found among prioritised Lead SNP pairs by increasing our analytical and statistical stringency. First, we decreased our LD allowance between Lead SNP pairs from *r*^2^<0.8 down to *r*^2^<0.01 (Supplementary Figure S8), which yielded very similar enrichment results. This demonstrated that the observed enrichment of shared biological mechanisms within the same disease was unlikely to be merely the result of LD. Second, we reproduced our analysis using an alternate eQTL dataset derived from liver,^32^ which, despite being 12-fold smaller and calculated with a more stringent *P* value, demonstrated that the enrichment of shared biological process mechanisms was not confounded by tissue source (Supplementary Figure S9). Interestingly, in the liver eQTL data we were able to prioritise within-disease SNP pairs for hepatitis-B vaccine response and primary biliary cirrhosis, which both involve liver as a target organ. These suggest tissue-specific patterns of expression may be having important roles in addition to common patterns. Third, within-disease SNP pairs have more similarities and mRNA overlap than SNP pairs that span across distinct diseases even beyond the most rigorously prioritised results. Using all inter–inter and inter–intra Lead SNP pairs and relaxing *P* values by one or two orders of magnitude, we continue to see the data asymmetry with the majority of significant *P* values in the same-disease results (left skew in Q–Q plots, LD *r*^2^<0.01; Supplementary Figure S10 and Supplementary Methods). Fourth, we performed the enrichment analysis again using an alternate reference human genome annotation, which includes coordinates for microRNA and lncRNA (GENCODE^33^ version 19; best OR=25.4, *P*=6.4×10^−6^ ) to establish that our results were not the result of miscategorising SNPs within this region as intergenic (Supplementary Figure S11). Fifth, similar enrichment results were observed by applying a *P*<0.05 cutoff (OR=13, one-sided FET *P*=3.1×10^−5^). Overall, these controls demonstrated the approaches chosen for the pairwise comparisons and prioritisations were reproducible under multiple conditions. We additionally confirmed that the enrichment results were not driven by diseases that had few GWAS SNPs. On the contrary, more SNPs and more studies per disease increased the chance of yielding more SNP pairs with shared biological mechanisms (Supplementary Figure S12).

### GWAS-based evidence of non-additive synergistic genetic risk interactions among prioritised lead SNP pairs associated with bladder cancer and Alzheimer’s disease

On the basis of substantial evidence for shared mechanisms among prioritised Lead SNP pairs associated with the same disease, we hypothesised that a subset of SNPs could exhibit genetic interactions. Using independent data set of disease–SNP associations,^34,35^ we applied a multifactor dimensionality reduction method to detect and characterise non-additive genetic interactions^36,37^ among the Lead SNPs found *a priori* in the prioritised SNP pairs associated with bladder cancer (two pairs) and Alzheimer’s disease (six pairs). The multifactor dimensionality reduction analysis revealed significant synergistic interactions for two Alzheimer’s disease pairs and one of the bladder cancer pairs (Table 1). These results were significant after keeping the main effects constant and adjusting for multiple comparisons using permutation testing. In addition, SNP combinations showed evidence of synergistic effects using entropy-based measures of interaction information. This result showed that SNPs engage in cooperative or epistatic effects indicative of functionally similar mechanisms.

### Genetic interactions of Lead SNP pairs prioritised by shared biological mechanisms in a phenome-wide association study of RA

**Figure 4:**
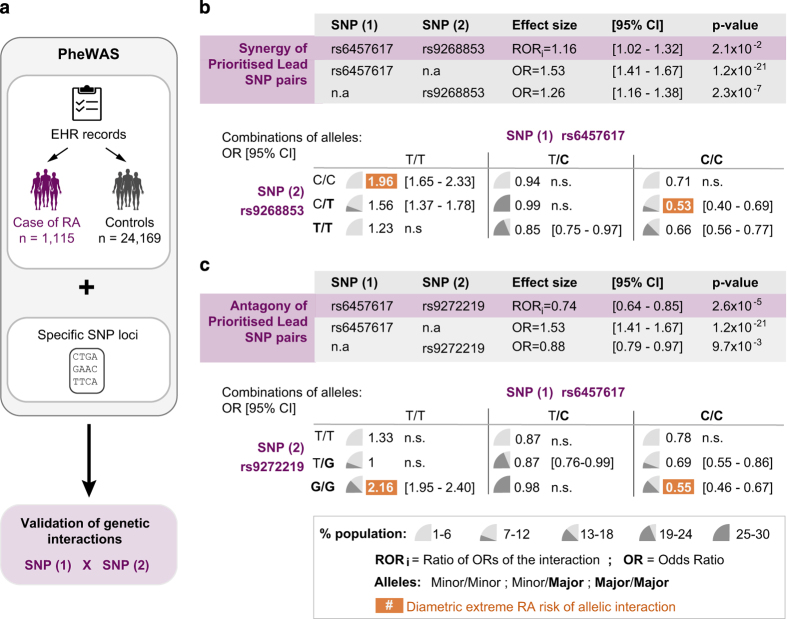
PheWAS illustrates genetic interactions in prioritised inter–inter Lead SNP pairs associated with rheumatoid arthritis. Non-additive genetic interaction of prioritised inter–inter Lead SNP pairs was confirmed in an independent population of 1,115 RA cases and 24,169 controls. (**a**) Overview of the PheWAS and genetic interaction validation process. (**b**) A synergistic effect was observed between unlinked (*r*^2^=0) SNP(1) *rs6457617* and SNP(2) *rs9268853*. (**c**) An antagonistic effect was observed between almost unlinked (*r*^2^=0.017) SNP(1) *rs6457617* and SNP(2) *rs9272219*. The upper parts of **b**, **c** provide insight into the effect size parameters of the logistic regression model. For example, genetic interaction is measured between two SNPs when the Ratio of OR of the interaction (ROR_i_) differs significantly from the value 1. Synergy corresponds to an increased ROR_i_, whereas antagony relates to its decrease. The combination of effect size parameters of each SNP taken alone (odds ratio; OR) with those of the interaction (ROR_i_) is required to estimate the OR associated with a specific set of minor and major alleles of both polymorphisms. The lower tables of **b**, **c** provide a systematic view of the specific OR and populations associated with each allelic combination of these interacting polymorphisms.

We next tested prioritised Lead SNP pairs associated with RA, using a PheWAS derived validation method for genetic interactions. SNPs were characterised in patients participating in the BioVU DNA biorepository^38^ project linked to an anonymous version of the Vanderbilt University Electronic Health Record (EHR), from which RA subjects were identified based on PheWAS (Figure 4a). We first confirmed that, as expected, each Lead SNP in these pairs was actually associated with RA in this independent data set (*P*<0.01). Using logistic regression incorporating the ratio of ORs for genetic interaction (ROR_i_), we further identified both SNP–SNP synergy and antagonism among the RA-associated prioritised Lead SNP pairs (Figure 4b,c). For example, the Lead SNP pair comprised of *rs6457617* and *rs9268853* exhibited synergistic genetic interaction (ROR_i_=1.16; *P*=0.021; Figure 4b). For these SNPs, we observed increased risk of RA (OR=3.4, *P*=6.6×10^−14^) when we compared the diametric extreme ORs of their alleles (Figure 4b). In contrast, the genetic interaction of Lead SNPs *rs6457617* and *rs9272219* displayed an antagonistic effect (ROR_i_=0.74; *P*=2.6×10^−5^; Figure 4c). Because of the antagonism, the homozygous major alleles for *rs9272219* alternatively increase or decrease the risk of RA when, respectively, combined with either the minor or major alleles for *rs6457617* (OR of diametric extremes=3.2, *P*=2.2×10^−16^; Figure 4c). The homozygous major alleles for *rs9272219* are associated with increased RA risk in the presence of the minor alleles for *rs6457617* (OR=2.16 versus OR≈1; Figure 4c), but they are associated with the lowest risk of RA in the presence of the major alleles for *rs6457617* (OR=0.55, *P*=7.2×10^−9^; Figure 4c).

### Interacting TFs and regulatory elements from ENCODE corroborate converged mechanisms prioritised between Lead SNPs

**Figure 5:**
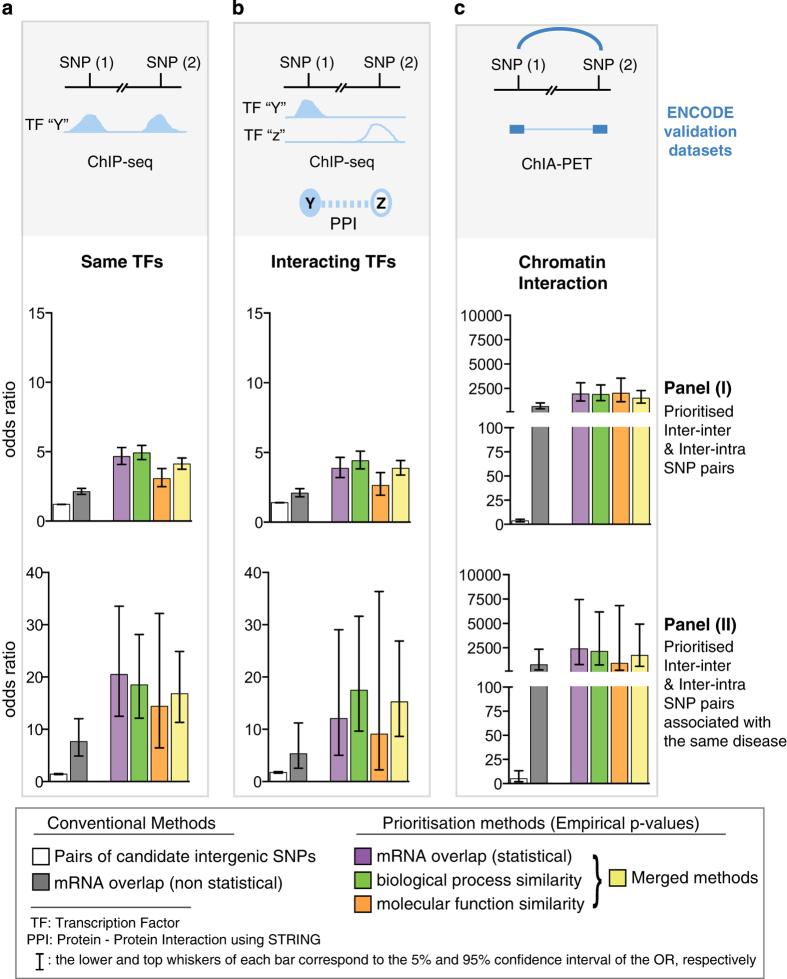
Prioritised inter–inter and inter–intra Lead SNP pairs are enriched in genomic regions sharing common ENCODE-derived transcription factors (TFs) and regulatory elements. ENCODE data were used to assess the propensity of prioritised inter–inter and inter–intra Lead SNP pairs to localise in regulatory regions with the same (**a**) TF(s) via ChIP-seq, (**b**) two distinct interacting TFs (ChIP-seq and protein–protein interactions, PPI) and (**c**) long-range chromatin interaction properties (ChIA–PET). Enrichment of inter–inter and inter–intra Lead SNP pairs (odds ratios with 95% confidence, *y* axis) in regions sharing common regulatory properties were evaluated between (i) prioritised and non-prioritised Lead SNP pairs (Panel (I)), (ii) prioritised Lead SNP pairs in the same disease and across-diseases (Panel (II)). Greater ORs are observed in disease-specific SNP pairs (Panel (II) compared with Panel (I)); ORs range from 2.6 to 1998.9 (3.4×10^−136^⩽*P*⩽5.3×10^−8^) in Panel (I) and 9.1 to 2249.9 (3.5×10^−22^⩽*P*⩽2.1×10^−2^) in Panel (II). Candidate inter–inter and inter–intra SNPs considered for the enrichments were associated with mRNAs by eQTL with *P*⩽10^−4^ (mRNA overlap; grey bars). Stringent prioritisations using empirical computations were performed on mRNA overlap (mauve bars), biological process similarity (green bars), molecular function similarity (orange bars) and in combination (merged methods; yellow bars). Enrichments of SNP pairs were performed using Fisher’s exact test among all pairwise combinations of NHGRI disease-associated SNPs. Potential causal SNPs represented by the Lead SNPs in the pairs were included in this regulatory function study and were taken from RegulomeDB (Materials and Methods).

We further hypothesised that intergenic disease-SNPs located in genomic regions surveyed for DNA–protein interactions and *cis*-element activities would enable us to identify and characterise the molecular regulation of prioritised biological mechanisms. We incorporated ENCODE-derived biochemical assays^18^ into our study to explore three regulatory properties that Lead SNPs within each pair may share: (i) distinct SNP regions harbouring the same TFs (ChIP-seq; Figure 5a), (ii) SNP regions with distinct interacting TFs (ChIP-seq and protein–protein interaction; Figure 5b) or (iii) SNP regions that physically interact via specific proteins (ChIA–PET; Figure 5c). Using RegulomeDB,^39^ we also extended the study of Lead SNPs by including ENCODE-derived annotations of SNPs in strong LD (LD SNPs; *r*^2^⩾0.8) with each SNP within a Lead SNP pair. These Lead or LD SNPs may have a causative effect and/or contribute similarly to disease pathogenesis. By combining annotations, we showed Lead SNP pairs with shared biological mechanisms are more likely enriched in regions with common regulatory properties than non-prioritised SNP pairs (Figure 5, Panel (I)). Among 3,870 inter–inter and inter–intra lead SNP pairs, we recovered 473 pairs that share genomic regions with same TFs (441 pairs), interacting TFs (223 pairs) or (31 pairs) long-range interactions. Moreover, we demonstrated that the surveyed regulatory properties were enriched among 26 prioritised inter–inter and inter–intra SNP pairs associated with the same disease, but not across distinct diseases (Figure 5, Panel (II)).

We observed substantial enrichment of prioritised inter–inter and inter–intra Lead SNP pairs in regulatory and interacting genomic regions across the three imputed biological mechanisms predicted by our methods when compared with conventional approaches, with one exception out of 12 comparisons (95% interval whiskers, Figure 5, Panel (I)). Conventional eQTL-related methods involved identifying (i) any pair of Lead SNPs with at least one associated mRNA (*P*⩽10^−4^) or (ii) straightforward (non-statistical) overlap of mRNA(s) associated with each Lead SNP of a pair. Notably, the enrichment was generally more pronounced for prioritised SNP pairs associated with the same disease, as indicated when comparing the whiskers of each prioritisation method in Panel (I) to its counterpart in Panel (II) (nonoverlapping whiskers, Figure 5). We observed at least a threefold increase in the OR for prioritised Lead SNP pairs associated with the same disease using the ENCODE ChIP-seq of transcription factors (Figure 5a,b). In addition, ChIA–PET-based analysis revealed further enrichment (OR>2,500) of SNPs co-localising with genomic regions undergoing long-range interactions mediated by chromatin-modelling DNA binding proteins of CTCF or catalysers of DNA transcription, such as RNA polymerase II.^40,41^ This remarkable increased enrichment is related to the nature of the ChIA-PET assays, which capture the regulatory network of transcriptional and chromatin structural activities that mirror many putative regulatory associations computed from SNPs with expressed quantitative traits (Figure 5c). The ORs improved across every prioritisation method and each of the ENCODE validation data sets when computed at an eQTL cutoff of *P*⩽10^−6^ (OR>9,000, one-sided FET *P*=1.2×10^−11^), rather than using a fixed eQTL cutoff of *P*⩽10^−4^ as performed in our initial enrichment analysis illustrated in Figure 5. In addition, ORs remain significant but slightly less when prioritising the Lead SNP pairs at the anticonservative nominal *P*<0.05 (OR=896.7, one-sided FET *P*=3.5×10^−11^). An even more stringent LD cutoff of *r*^2^<0.01 (Supplementary Figure S13) yielded comparable ORs to those from LD *r*^2^<0.8, suggesting that the convergent regulatory mechanisms between prioritised SNPs were unlikely to be the result of linkage disequilibrium. These results support the notion that SNPs related to the same disease that affect same gene expression and similar biological mechanisms are often correlated with similar functional *cis*- and/or *trans*-regulatory elements that often engage in long-range chromatin interactions such as enhancer–promoter and enhancer–enhancer interactions.

## Discussion

Here we developed a computational method that combines different levels of genomic information (GWAS, eQTL and ENCODE) and knowledge base of gene annotations (GO) to impute biological effectors of SNPs derived from their shared biological downstream mechanisms. We showed that intergenic and intragenic SNPs predisposing an individual to the same disease most likely affect expression of the same mRNAs, mRNAs involved in similar biological pathways or governed by similar regulatory mechanisms. Among the 2 million surveyed SNPs, and at stringent cutoff of FDR<0.05, our prioritisation methods unveiled (i) 3,870 prioritised inter–inter and inter–intra Lead SNP pairs among 312 diseases that share at least one of the imputed biological mechanisms, (ii) about one third of the SNP pairs were selectively identified by at least two prioritisation methods, (iii) 80 disease-specific inter–inter and inter–intra Lead SNP pairs with shared mechanisms among 32 diseases and (iv) 473 prioritised inter–inter and inter–intra SNP pairs in regions with common regulatory properties, among which 26 inter–inter and inter–intra pairs are of the same disease. We further validated a subset of these predictions with non-additive genetic risk interactions in an independent association data set for three human diseases as well as with ENCODE-informed validations of regulatory elements. According to ENCODE regulatory data, prioritised Lead SNP pairs were also enriched for similar regulatory elements (enhancer, promoter and TFs binding sites) and were involved in the same chromatin long-range interactions. These results showed that intergenic and intragenic SNPs share disease effects through shared functionality at different level of scale of biology.

Using mRNA overlap, previous study of Fehrmann *et al.* recovered seven disease-specific unique SNP pairs (trans-eQTLs) at FDR<0.05 among four diseases that shared mRNAs with converged biological pathways.^42^ We showed that our prioritisation methods were able to recover substantially more predictions by GO–BP and GO–MF similarity to identify shared mechanisms for SNP pairs without mRNA overlap. This suggests that we have successfully enriched for those intergenic SNPs that reveal a functional impact on disease pathology, although identifying which GWAS SNPs are truly causal rather than associated or perhaps even spurious is a task beyond the scope of this study. If all GWAS SNP inputs could be refined to the causative variant, then we expect to see a significant increase in functional overlap across each disease. Another limitation of our approach is that it relies heavily on biased GO knowledge annotations that are not designed to uncover non-canonical and poorly characterised biological mechanisms. We also observed a high number of prioritised Lead SNP pairs related to immune related loci (e.g., MHC/HLA) and their downstream activities, which is consistent with the well-described role for HLA and inflammatory processes in many complex diseases, including those studied by GWAS. It is also possible that these are over-represented here due to the nature of the lymphoblastoid cell lines used for eQTL studies and their context-specific stimulations linked to particular diseases.^14,42^ Although many studies have reiterated such observations, neither consensus nor guidelines regarding the optimal cell lineage from which to derive eQTL associations that are most qualified for imputing disease-specific pathogenesis has been established. However, numerous eQTL and genomic annotation-based studies showed that analysing multiple cell types^25,43–45^ could uncover novel mechanisms and biomodules that explain organs or tissue system implications in overall disease pathology. Future directions for identifying biomodules from SNPs could involve the use of unbiased gene sets such as those obtained by co-expression networks^46,47^ or computational gene similarity measures.^48^ These prioritisation statistics can also be applied in a targeted manner to a given disease rather than the GWAS catalogue as a whole, where a specific disease-relevant eQTL dataset may be obtained and less stringent nominal *P* values can be used for biomodule discovery without as much need for multiple testing correction. Further investigation in this direction is supported by our independent prioritisation of SNP pairs associated with liver diseases (Primary biliary cirrhosis and Hepatitis B vaccine response) when using the liver eQTL data set. Finally, this current study was computationally intensive as the empirical resampling was conducted homogeneously across pairs. The algorithms can be optimised by conditioning the resampling according to SNP pairs and dynamically ending the resampling when *P* values observed are non-significant. These improvements should allow to investigate further the effect of eQTL derived from cell types more relevant to specific diseases, such as those available in Genotype-Tissue Expression data sets GTEx.^49^

Previous computational studies preferentially used ENCODE data sets as a seed to map SNPs to DNA regulatory elements with putative function and used the results to associate these SNPs qualitatively (literature curation) and quantitatively (gene set enrichment in knowledge bases or network models) to predict downstream biomolecular mechanisms.^23–25,50–52^ In contrast, our approach leverages ENCODE data to determine whether prior SNP-associated disease mechanism predictions share regulatory elements that might explain their convergent effects. New genome-wide regulatory annotations and quantitative trait loci analyses are now increasingly available such as those derived from chromatin accessibility and DNA methylation patterns of non-coding regions. Approaches relying on similarity of biological mechanisms could be systematically applied to these growing genomic data sets and further inform how common polymorphisms are involved in transcriptional or post-transcriptional mechanisms underlying the regulatory and cellular networks of disease progression.

This study highlights the significance of mechanistic similarities for uncovering additional interacting downstream effectors of intergenic SNPs predisposing individuals to the same disease. Identifying and understanding mechanisms of disease can not only inform biology but also provide insight in identifying candidate therapeutic targets. These results can be pursued for generating a comprehensive ‘roadmap’ of disease mechanisms revealed by downstream effectors of intergenic SNPs.

## Materials and methods

Data sets/database are described below and in detail in Supplementary Figure S1 and Supplementary Table S2.

### eQTL association

Two eQTL association data sets were acquired from SCAN-DB. The bulk of this analysis was done using an eQTL data set of the lymphoblastoid cell lines,^26^ which consisted of 4,189,682 associations between 833,004 distinct SNPs and 11,860 mRNAs at *P*⩽10^−4^. Each SNP included for further study was matched to at least one eQTL transcript with a median of 2 transcripts per SNP (Supplementary Figure S3). The liver tissue eQTL dataset used for validation (Supplementary Methods; Supplementary Figure S9) was comprised of 314,545 associations between 139,814 SNPs and 19,641 mRNAs at *P*⩽10^−5^.^53^ Trans effect was defined as 4 M bp from SNP to target mRNA based on the original definition^54^ and dbSNP build 138^27^ and refSeq^55^ hg19 coordinates.

### National human genome research institute GWAS catalogue

The dataset comprises 7,236 associations between 574 diseases/traits with 6,432 unique Lead SNPs.^2^

### dbSNP

SNPs associated with human disease (National human genome research institute (NHGRI) GWAS catalogue) and mRNA expression (eQTL) were characterised as inter- or intragenic SNPs according to dbSNP (Build 138) definitions, which are based on RefSeq gene coordinates. Intragenic SNPs are located in regions whose boundaries extend 2 kb upstream of the transcription start site and 0.5 kb downstream of the terminator according to RefSeq.^55^ Intergenic SNPs are located between two intragenic regions.^27^

### GO annotations

GO annotations for human genes were retrieved from NCBI^28,56^ and used to associate mRNA (eQTL) with molecular function (GO–MF) and biological process (GO–BP) terms. The database consisted of GO–MF and GO–BP annotations for 11,774 and 9,717 distinct genes (mRNAs), respectively.

### STRING and protein–protein interactions

The STRING v9.1 database was used to determine PPIs among TFs.^57^ Only interactions between distinct TFs that scored ⩾0.9 were included in the enrichment analyses (Figure 5).

### ENCODE data set

This data set provides DNA element annotations of the human genome based on various biochemical assays such as ChIP-seq, DNase-seq and RNA-seq.^18^ We leveraged two types of ENCODE data for the enrichment analyses: (i) combined data set of TF binding sites (TFBS-Clustered) comprising ChIP-seq of 148 TFs across 95 cell lines and (ii) three ChIA-PET data sets (Pol2, CTCF and ESR1) with data collected from cell lines, K562, HeLa, MCF-7, HCT-116 and NB4.

### Prioritisation of SNP pairs

We included 2,358 SNPs (Supplementary Data S1; 1,092 intergenic SNPs) associated with both disease risk and gene expression for a pairwise analysis. We used the HapMap CEU LD data set to determine Lead SNP pairs with LD of *r*^2^<0.8 or *r*^2^<0.01.^58^ SNP pairs in strong LD (LD, *r*^2^⩾0.8) were excluded from the study. Among the remaining pairs, we focused on inter–inter and inter–intra Lead SNP pairs (2,039,944) with at least one intergenic SNP. We then employed three methods based on a high-throughput computing system to prioritise biological mechanisms shared among SNP pairs: (i) mRNA overlap, (ii) molecular function similarity and (iii) biological process similarity. These prioritisations were controlled by permutation resampling of scale-free networks.^3,30^

### Computed shared mechanisms: mRNA overlap and semantic biological similarity of SNP pairs

Prioritisation by mRNA overlap measured the number of shared mRNAs between two SNPs; typically, the number of shared mRNAs was directly related to mRNA overlap. We reported both non-statistical (any overlap) and statistical (prioritised by permutation resampling) types of mRNA overlap. Prioritisation by biological similarity was based on GO annotations of mRNA molecular functions or biological processes associated with the SNPs within each pair. Briefly, as every SNP within a pair could be associated with multiple mRNAs, and every mRNA could be associated with multiple GO terms, we performed three steps to impute biological similarly between two SNPs. First, we calculated the information theoretic semantic similarity (biological similarity) among GO terms^59^ as described in our previous work.^29^ We then computed the biological similarity of each pair of mRNAs within an SNP pair based on the average biological similarity of GO term pairs associated with the two mRNAs.^7,60^ Finally, we developed an algorithm to impute the biological similarity of an SNP pair based on the average biological similarity of mRNAs associated with the two SNPs as the following ‘Equation (1)’.

(1)SNP_ITS(s1,s2)=∑gi∈G(s1)maxgj∈G(s2)(GENE_ITS(gi,gj))+∑gj∈G(s2)maxgi∈G(s1)(GENE_ITS(gi,gj))|G(s1)|+|G(s2)|

where SNP *s*_*1*_ was associated with a set of mRNAs *G(s*_*1*_), and |*G(s*_*1*_)| is the cardinality of the set *G(s*_*1*_), similarly for *s*_*2*_. The GENE_ITS_ is the biological similarity of two mRNAs^7,60^ (details in Supplementary Methods). The SNP_ITS provides a score that ranges from 0 to 1; a value of 1 indicated two SNPs with common GO–MFs or GO–BPs, and a value of 0 corresponded to two SNPs with unrelated GO–BPs or GO–MFs.

### Permutation resampling for prioritisation of computed shared mechanisms

The three prioritisation methods were subjected to stringent statistical measurements to filter the relationship between two SNPs that could be observed by chance (Supplementary Methods). In contrast to straightforward resampling methods, we performed permutation resampling with node-degree conservation on the entire eQTL association network (SNP–mRNA). Thus, we could control for the distinct probability of each SNP and mRNA, given original eQTL association network’s topology. For each empirical permutation, the number of mRNAs associated with each SNP (SNP node degree) and the number of SNPs associated with each mRNA (mRNA node degree) conserved the same cardinality of connections as in the original eQTL data set. For each SNP pair, a *P* value was calculated as the proportion of empirical permutations (frequency among 100,000 times) with equal or greater strength of overlap or biological similarity than those observed. We then adjusted for multiplicity using the Benjamini–Hochberg FDR procedure independently for each of the three prioritisation methods using the *p.adjust* function in R software (http://www.r-project.org/). Prioritised SNP pairs were those yielding sufficient statistical significance using any of the prioritisation methods.

### Computations

Approximately 20,000,000 core hours of high-throughput computations were conducted on the *Beagle* GLOBUS^61,62^ computing infrastructure housed in a Cray XE6 Supercomputer of the Computation Institute at the Argonne National Laboratory with peak performance of 151 teraflops generated by 17,424 compute cores (http://beagle.ci.uchicago.edu/).

### Enrichment analysis of disease mechanisms among prioritised SNP pairs

We performed an enrichment analysis to assess whether shared mechanisms (mRNA overlap, GO–MF/GO–BP similarity) were more likely found among SNP pairs related to the same disease than those across distinct diseases. Therefore, we dichotomised all SNP pairs into those associated with the same disease and those associated with distinct diseases based on the NHGRI GWAS catalogue. We then performed SNP pair enrichment by calculating ORs and *P* values according to the following contingency table: (same disease versus across-disease SNP pairs)×(prioritised versus non-prioritised SNP pairs) using Fisher’s exact test in R. We also performed enrichment tests at different *P* value cutoffs of eQTL associations (⩽10^−4^ to ⩽10^−6^) from which the number of mRNAs associated with each SNP served as a threshold for calculations (⩾1, ⩾3 and ⩾5 mRNAs per SNP).

### Enrichment analysis of common regulatory properties among prioritised SNP pairs

Pairs were prioritised according to computed shared mechanisms as described above. For each mechanism, we determined whether prioritised SNP pairs were enriched in genomic regions with common regulatory properties: (i) same TF binding sites, (ii) interacting TFs and (iii) long-range chromatin interactions. Specifically, we leveraged ENCODE data sets to attribute DNA element annotation(s) to each SNP of the prioritised pairs, such as TF binding sites (ChIP-seq data) and/or anchored regions with long-range interactions (ChIA-PET) data. We extended the regulatory annotation of the Lead SNPs to SNPs in strong LD (*r*^2^⩾0.8) with each Lead SNP of a pair. RegulomeDB^39^ was used to determine Lead SNPs in strong LD (LD SNPs; *r*^2^⩾0.8) for which ENCODE-derived functional annotations were available. The first enrichment analysis assessed whether prioritised SNP pairs are more likely than non-prioritised pairs to be enriched in regions sharing common regulatory properties using the following contingency table: (same regulatory properties versus different regulatory property of Lead SNP pairs)×(prioritised versus non-prioritised Lead SNP pairs). We performed the second enrichment analysis to determine whether prioritised SNP pairs related to the same disease are more likely to share common regulatory properties than those associated with distinct diseases using the contingency table: (same disease and regulatory properties versus distinct diseases and/or different regulatory property Lead SNP pairs)×(prioritised versus non-prioritised Lead SNP pairs). We included a control in which SNP pairs were calculated from every possible combination of SNPs with an eQTL association. All Lead SNP pairs derived from the NHGRI GWAS catalogue were used as the background, and enrichment analyses were performed on SNP pairs derived from eQTL associations with *P*⩽10^−4^. Bar graphs were generated using Prism v.6 (GraphPad Software Inc, La Jolla, CA, USA).

### GWAS-based detection of epistatic effects among mechanism-anchored prioritised Lead SNP pairs

Per our *a priori* hypotheses, prioritised intergenic Lead SNP pairs associated with bladder cancer (BC) or Alzheimer's disease (AD) were considered for genetic interactions in GWAS (BC: *rs9642880–rs1495741 and rs8102137–rs1014971; AD: rs7081208–rs9331888, rs17511627–rs9331888, rs3818361–rs4509693, rs381836–rs7081208, rs4509693–rs753129 and rs4509693–rs6656401*). We first applied the multifactor dimensionality reduction machine-learning method^36^ for modelling the joint effects of the Lead SNP pairs. The multifactor dimensionality reduction approach was implemented using 10-fold cross-validation for estimating generalisability, followed by a 1,000-fold permutation test to determine statistical significance and to address multiple testing issues. In addition, we applied the explicit test of epistasis, which uses permutation testing to determine statistical significance of interaction effects while holding the main effects constant.^63^ An entropy-based information gain approach^64,65^ was used as an additional method for interpreting the statistical pattern of epistasis. The BC GWAS included 3,532 cases and 5,119 controls from the Cancer Genetic Markers of Susceptibility for Bladder Cancer study,^34^ which is available from dbGaP (accession: phs000346.v1.p1). The AD GWAS included 529 cases with mild cognitive impairment or AD and 204 controls from Phase I of the Alzheimer’s Disease Neuroimaging Initiative,^35^ also available from dbGaP (accession phs000219.v1.p1).

### PheWAS identification of genetic interactions among mechanism-anchored prioritised Lead SNP pairs

Each RA-associated prioritised inter–inter and inter–intra Lead SNP pair was considered for SNP–SNP interactions using a data set selected from the Vanderbilt University EMR-linked DNA biobank (BioVU).^38^ To identify RA case–controls cohort from the EHR, we utilised previously developed PheWAS case–control definitions for RA that can reproduce known genetic associations.^66,67^ From a population of approximately 36,000 individuals with extant Illumina Human Exome chip genotype data in the deidentified Vanderbilt University clinical data warehouse linked to BioVU,^38^ we identified 1,115 RA cases and 24,169 controls (Supplementary Table S3). Cases had at least two ICD-9-CM billing codes (http://www.cms.gov/Medicare/Coding/ICD9ProviderDiagnosticCodes/codes.html) specific to RA (714.0, 714.1, 714.2 or 714.81) on different days. Controls were selected among patients with no RA or related diagnoses (e.g., juvenile idiopathic arthritis, psoriatic arthritis) reported in their billing history according to the PheWAS approach. Individuals with RA noted on a single day were excluded, as these cases often have poorer positive predictive value.

For each patient, we had previously extracted DNA and genotype data for 233,605 SNPs with <5% missing data using the Illumina Human Exome 12v.1 array. Genotypes were quality controlled for call rate (>95%), minor-allele frequency (>1%) and identity by descent to remove related individuals. Among these genotyped SNPs, three prioritised Lead SNP pairs (involving SNPs ‘alleles’ *rs6457617-‘T/C’, rs9272219-‘T/G’ and rs9268853-‘C/T’*) associated with RA were available for calculations. Only individuals identified from European ancestry by Structure^68^ were used in the analysis, resulting in 29,731 individuals before case and control selection. All association analyses were completed with PLINK v1.07^69^ using logistic regression adjusted for age and sex and assuming an additive genetic model. Interaction analyses were also performed on the second SNP of each pair and included an SNP–SNP interaction term (ROR_i_). Interactions between specific alleles of Lead SNP pairs were analysed by Fisher’s exact test. ORs of allelic combination effects associated with RA and their 95% confidence intervals were reported using PLINK v1.07. Submission to dbGaP of RA genotypes and phenotypes of the present PheWAS study is in process.^70^

### Network of predicted mechanisms shared by disease-associated prioritised Lead SNP pairs

On the basis of the disease-specific results of this study, a global network of functional annotations was constructed that comprises biological molecules and their relationships across the three prioritisation methods (SNP–mRNA eQTL, prioritised SNP–SNP association and computed SNP–GO–SNP association). Disease-specific networks curated to highlight overlap and similarity of mechanisms found among prioritised Lead prioritised SNP pairs associated with RA. Networks were visualised using Cytoscape.^71^ Technical details regarding network construction are found in Supplementary Methods.

### Original software

Source code used in this manuscript has been made freely available at http://www.lussierlab.org/publications

Supplementary Table S4 presents key concepts and abbreviations.

## Figures and Tables

**Table 1:** Non-additive genetic interaction of prioritised inter–inter and inter–intra Lead SNP pairs validated in independent GWAS studies

*Disease*	*Prioritised SNP pairs*	*SNPs with synergistic effects*	*Entropy* P*-value*
Alzheimer’s	rs4509693–rs753129 (chr10, inter) (chr4, inter) rs7081208*–rs9331888* (chr10, FRMD4A) (chr8, CLU, MIR6843)	rs4509693–rs753129–rs7081208*	0.046
Bladder cancer	rs8102137–rs1014971 (chr19, inter) (chr22, inter)	rs8102137–rs1014971	0.039

Abbreviations: eQTL, expression quantitative trait loci; SNP, single-nucleotide polymorphism.

Entropy-based *P* values correspond to the observed statistical pattern of epistasis. SNP rs4509693 was associated with both cis and trans-eQTLs, but all other eQTL relationships were trans. Asterisks are used to indicate intragenic SNPs with host gene listed below.
