# Supplementary material for: Integrative genomics analyses unveil downstream biological effectors of disease-specific polymorphisms buried in intergenic regions
Source: NPJ Genom Med. 2016 Apr 27;1:16006–. doi: 10.1038/npjgenmed.2016.6 (PMC4966659; doi:10.1038/npjgenmed.2016.6)
Supplement: Supplementary Information [file npjgenmed20166-s1.pdf]

Li et al. Supplementary Information

Integrative genomics analyses unveil downstream biological effectors of disease-specific polymorphisms buried in intergenic regions

	Page
**I- Supplementary Methods**	
A) Calculation of SNP similarity	2
B) Permutation methods	3
C) Q-Q plot analysis	4
D) Disease Network	4
**II- Supplementary Figures**	
Supplementary Figure 1: Workflow	6
Supplementary Figure 2: Violin plots of the input	8
Supplementary Figure 3: Circos plot of the results	9
Supplementary Figure 4: Genomic distance and LD of the results	10
Supplementary Figure 5: Results of inter-intra Lead SNP pairs	11
Supplementary Figure 6: Results of intra-intra Lead SNP pairs	12
Supplementary Figure 7: Full network of Rheumatoid arthritis	13
Supplementary Figure 8: Results of unlinked SNPs	15
Supplementary Figure 9: Liver results	16
Supplementary Figure 10: Q-Q plots	17
Supplementary Figure 11: GENCODE results	19
Supplementary Figure 12: Bias analysis	20
Supplementary Figure 13: ENCODE mechanisms of unlinked SNPs	21
**III- Supplementary Tables**	
Supplementary Table 1: Prioritized Lead SNP pairs	22
Supplementary Table 2: Data source	23
Supplementary Table 3: RA cohort	25
Supplementary Table 4: Abbreviations	26

I- Supplementary Methods

A) Calculation of SNP similarity. This calculation requires three steps (A1, A2, and A3).

A1. Information Theoretical Semantic Similarity of two Gene Ontology (GO) terms (ITS). We used Lin's method (Lin 1998) to calculate the ITS of two GO terms, t_1, t_2. This computes their similarity regarding ontology topology (e.g., two biological processes or two molecular function terms). It was defined (**Equation 1**) as the ratio of the information content (*ic*) of the minimal common ancestor *mca* of the two terms ($ic(ms,t_1,t_2)$) over the average information content of these two terms ($[ic(t_1)+ic(t_2)]/2$).

$$ITS(t_1,t_2) = \frac{2 * ic(mca(t_1,t_2))}{ic(t_1)+ic(t_2)} \qquad (1)$$

The information content *ic* of a GO term *t* was defined by the negative logarithm of the probability calculated as the ratio of (i) the number of terms in the ontology subgraph rooted at term *t:* ($\Psi(t)$); divided by (ii) the total number of terms in the Gene Ontology category rooted at the topmost ancestor *r:* ($\Psi(r)$) (**Equation 2**).

$$ic(t) = -\log\left(\frac{|\Psi(t)|}{|\Psi(r)|}\right) \qquad (2)$$

where $\Psi(t)$ represents the subgraph of GO terms rooted at GO term *t,* and $|\Psi(t)|$ is the cardinality of the subgraph.
This measure of ITS (**Equation 1**) value is thus scaled between 0 and 1, where 1 is a complete overlap of subgraph between two GO terms. For more details and examples, please refer to our previous papers (Tao et al. 2007; Li et al. 2012; Regan et al. 2012).

A2. Information theoretical similarity of two mRNAs (GENE_ITS): We used a conventional method to calculate the similarity (*GENE_ITS)* of two mRNAs (**Equation 3**). For the purpose of this calculation, we use the genes from which each mRNA was transcribed and thus do not mention mRNA in the calculations. In GO, each gene may be annotated to one or multiple Gene Ontology terms, noted as the "Set of GO terms of gene x" or simply "$T(g_x)$". The similarity between gene 1 (g_1) and gene 2 (g_2) is measured using the semantic similarity (**Equation 1**) between the set of GO terms associated to gene 1 ($T(g_1)$) and those associated to gene 2 ($T(g_2)$). Precisely, for a specific GO term (t_i) associated to gene 1, we calculate its GO similarity score ($ITS(t_i, tj)$) to every GO term (tj) associated to gene 2 ($tj \in T(g_2)$) and retain the maximum value among them. This is repeated for each GO term associated to gene 1. Next, the converse was calculated for each GO term associated to gene 2. Then, we calculated the average of all these maximum similarity scores to obtain the similarity of the two genes, noted as $GENE\_ITS(g_1,g_2)$. The *GENE_ITS* was formally denoted as follows:

$$GENE\_ITS(g_1,g_2)=\frac{\sum_{t_i\in T(g_1)}\max_{t_j\in T(g_2)}(ITS(t_i,t_j))+\sum_{t_j\in T(g_2)}\max_{t_i\in T(g_1)}(ITS(t_i,t_j))}{|T(g_1)|+|T(g_2)|} \quad (3)$$

where gene g_1 was annotated to a set of GO terms, $T(g_1)$, and $|T(g_1)|$, is the cardinality of the set $T(g_1)$. The *GENE_ITS* provides a score that ranges from 0 to 1, where *GENE_ITS* of 0 corresponds to two genes with no similar GO annotations and *GENE_ITS* of 1 corresponds to two genes with the same GO annotations. See Pesquita at al. (Pesquita et al. 2009) and our previous paper (Regan et al. 2012) for more details about this widely-used semantic measure.

A3. Novel Semantic biological similarity of two SNPs using eQTL associations (SNP_ITS): We developed a new method to calculate the biological similarity of two SNPs using their SNP-mRNA eQTL associations (or *SNP_ITS* for short) (**Equation 4**). The similarity between SNP 1 (s_1) and SNP 2 (s_2) is measured using the semantic similarity (**Equation 3**) between the set of mRNAs associated to SNP 1 ($G(s_2)$) and those associated to SNP 2 ($G(s_2)$). Precisely, for a specific mRNA (g_i) associated to SNP 1, we calculate its similarity score ($GENE\_ITS(g_i, g_j)$) to every mRNA (g_j) associated to SNP 2 ($g_j \in G(s_2)$), and retain the maximum value among them. This is repeated for each mRNA associated to SNP 1. Then the converse is calculated for each mRNA associated to SNP 2. Then, we calculated the average of all the maximum similarity scores to obtain the similarity of the two SNPs, noted as $SNP\_ITS(s_1, s_2)$. The *SNP_ITS* of two SNPs was formalized as follows:

$$SNP\_ITS(s_1,s_2)=\frac{\sum_{g_i\in G(s_1)}\max_{g_j\in G(s_2)}(GENE\_ITS(g_i,g_j))+\sum_{g_j\in G(s_2)}\max_{g_i\in G(s_1)}(GENE\_ITS(g_i,g_j))}{|G(s_1)|+|G(s_2)|} \quad (4)$$

where SNP s_1 was associated to a set of mRNAs $G(s_1)$, and $|G(s_1)|$ is the cardinality of the set $G(s_1)$, similarly for s_2. The *SNP_ITS* provides a score that ranges from 0 to 1, where *SNP_ITS* of 0 corresponds to two SNPs with neither similar mRNA annotations nor similar GO annotations. *SNP_ITS* of 1 corresponds to two SNPs with the same mRNA annotations or the same number of mRNAs each having the same GO annotations.

B) Permutation methods. eQTL associations were selected by cutoffs of p-values between 10^{-4} and 10^{-6} ($p \le 10^{-4}$, $p \le 5 \times 10^{-5}$, $p \le 10^{-5}$, and $p \le 10^{-6}$) and SNP node degree (ND) threshold between 1 and 5 (ND≥1, ND≥3, and ND≥5). Each eQTL dataset was regarded as a bipartite network consisting of SNP and mRNAs. A conservative permutation resampling that keeps the node degree of each specific SNP and specific mRNA constant (as observed) was conducted for each dataset (100,000 permutations). By this means, the permutation resampling matched the probability of a SNP or mRNA connected in the bipartite (from 1/#SNPs to 1 for SNPs and from 1/#mRNAs to 1 for mRNAs). Three types of empirical permutation were conducted: mRNA

overlap, GO biological process, and molecular functions. The one for mRNA overlap was directly permutated from the eQTL dataset with respect to a p-value cutoff and a node degree threshold. For GO biological process and molecular functions, the genes without respective GO annotations were filtered out before permutation, and the subsequent calculation of biological semantic similarity and significance were based on the filtered ones, by which potential biases from incomplete annotation could be controlled. The same set of permutations was used for calculation of GO biological process similarity of SNP pairs and specific overlapping GO biological process terms in the SNP pairs. The same rule was applied to calculations for GO molecular function. The permutations were conducted in supercomputers (specifically beagle) or clusters using MPI parallel computing.

C) Q-Q plot analysis. Quantile-quantile (Q-Q) plot was employed to show the distribution difference of SNP pair measures between the pairs of SNPs associated with the same complex diseases and those with different diseases. Two types of measures were conducted: FDR of mRNA overlap and FDR of mRNA similarity (GO-BP). To show the relative trend for SNP pairs derived from different eQTL strengths, multiple Q-Q plot curves were assembled in one panel, such as different p-value cutoffs of eQTL associations ($p \leq 10^{-4}$, $p \leq 10^{-5}$, and $p \leq 10^{-6}$), and different SNP node degree (ND) thresholds (ND≥1, ND≥3, and ND≥5). The Q-Q plot curves derived by multiple eQTL cutoffs were generated by the *"qqplot"* function in R software and the output of the function were extracted and plotted with customized shapes and colors to distinguish these curves. Since the p-values of SNP pairs were derived from empirical permutations (e.g. 100,000 times), they were truncated at the minimal observable p-values (10^{-5}) and manually set as 90% of that (e.g. $9*10^{-6}$), with corresponding FDR truncated accordingly. The axes of the figure, each for one group of SNP pairs, were shown in negative $\log_{10}$ scale ($-\log_{10}$FDR) to better visualize the pattern. In addition, Mann-Whitney U test ("wilcox.test" function in R) was performed to evaluate the overall mean between the two groups of SNP pairs of each curve, using FDR values directly rather than negative log values. The p-value result of each U-test was shown along with its correspondingly Q-Q plot curve.

D) Disease Network: *assessment of the GO term overlaps significance within a SNP pair.* This method focuses on assessing the significance of an overlap of GO terms within a SNP pair (SNP-GO-SNP triplet). It allows explaining which functional relationships are more likely to relate two SNPs of prioritized SNP pair. The method requires four steps. First, all Lead SNPs in the studied SNP pairs were related to mRNA by eQTL associations from the SCAN database. Second, each of these associated mRNAs was related to each of their corresponding GO terms using the Gene Ontology Annotations (molecular functions (GO-MF) biological processes (GO-BP)). We utilized the fully-derived GO annotation table, where each GO terms is directly associated to mRNAs and all mRNA associations to the ancestral GO terms. GO terms are organized in a hierarchical structure (directed acyclic graph). Third, GO terms were assigned to Lead SNPs via their respective mRNAs. For each SNP of the Lead SNP pair, a list of associated GO terms is calculated. Finally, from these associations, we can straightforwardly identify overlapping GO terms

between two Lead SNPs of the prioritized Lead SNP pair. Then, we impute the statistical significance (p-value) of those overlapping GO terms using empirical permutation resamplings (100,000 times) of SNP-mRNA associations based on eQTLs (eQTL p-value cutoff $\leq 10^{-4}$ for the reported results). Under these permutations, every mRNA is associated to the same number of SNPs and each SNP to the same number of mRNAs (constant node degree, constant number of total mRNA-SNP associations; see above section). However, the association of SNP-GO terms differs from the observed set through resampling. False Discovery Rate (FDR) was used to adjust for multiplicity. These calculations were performed separately for GO-BPs and GO-MFs to derive prioritized Lead SNP pairs and then combined. Each significant SNP-GO-SNP triplet (FDR<0.05) was considered as prioritized and therefore, as a putative shared mechanism for the prioritized SNP pair.

References

Li H, Lee Y, Chen JL, Rebman E, Li J, Lussier YA. 2012. Complex-disease networks of trait-associated single-nucleotide polymorphisms (SNPs) unveiled by information theory. *Journal of the American Medical Informatics Association* **19**: 295-305.

Lin D. 1998. An information-theoretic definition of similarity. In *15th International Conference on Machine Learning*, pp. 296-304, Madison, Wisonson, USA.

Pesquita C, Faria D, Falcao A, Lord P, Couto FM. 2009. Semantic Similarity in Biomedical Ontologies. *PLoS computational biology* **5**: e1000443.

Regan K, Wang K, Doughty E, Li H, Li J, Lee Y, Kann MG, Lussier YA. 2012. Translating Mendelian and complex inheritance of Alzheimer's disease genes for predicting unique personal genome variants. *Journal of the American Medical Informatics Association* **19**: 306-316.

Tao Y, Sam L, Li J, Friedman C, Lussier YA. 2007. Information theory applied to the sparse gene ontology annotation network to predict novel gene function. *Bioinformatics* **23**: i529-538.

Supplementary Figure 1

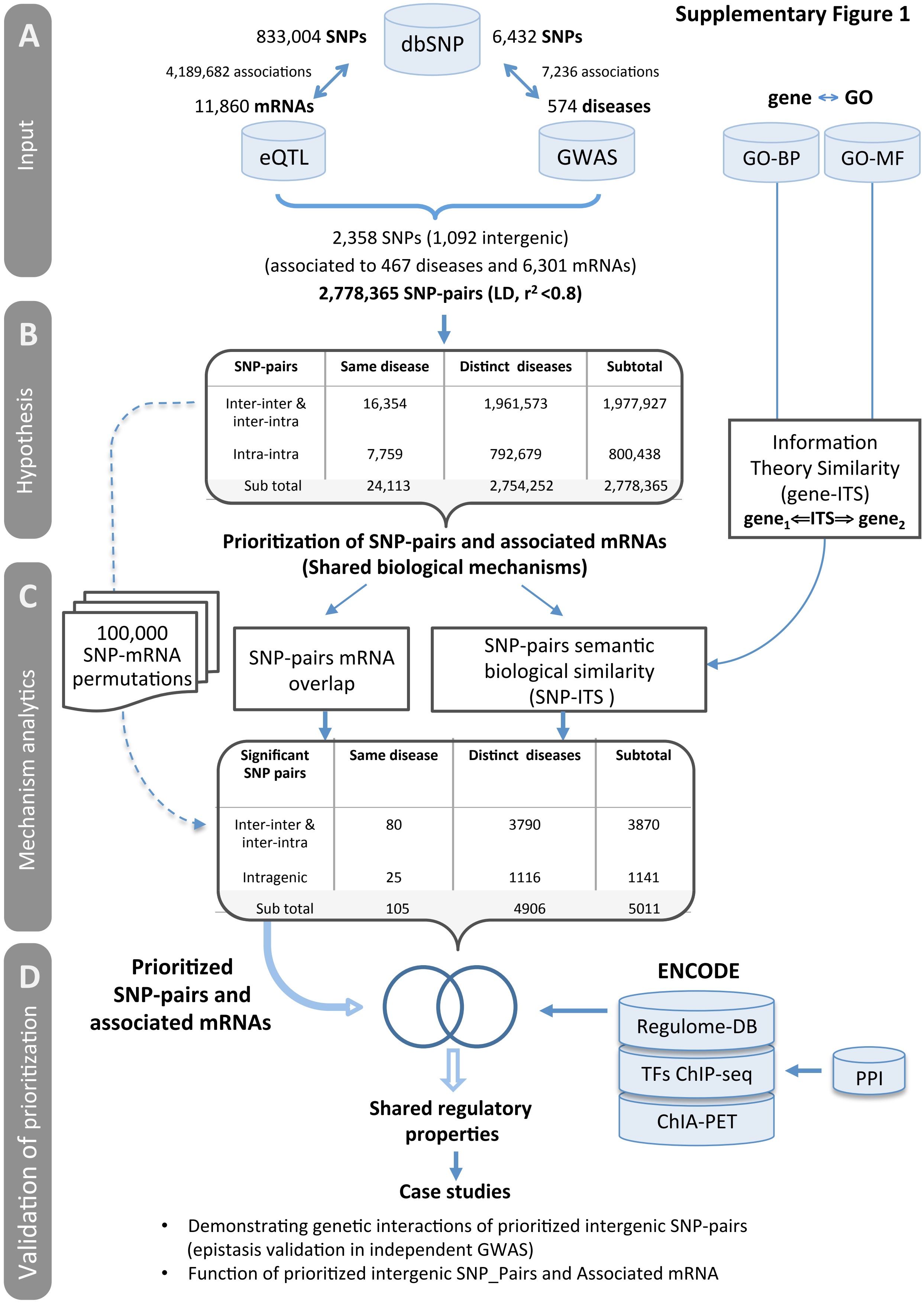

SNP-pairs	Same disease	Distinct diseases	Subtotal
Inter-inter & inter-intra	16,354	1,961,573	1,977,927
Intra-intra	7,759	792,679	800,438
Sub total	24,113	2,754,252	2,778,365

Significant SNP pairs	Same disease	Distinct diseases	Subtotal
Inter-inter & inter-intra	80	3790	3870
Intragenic	25	1116	1141
Sub total	105	4906	5011

Supplementary Figure 1. Workflow: identification of disease mechanisms shared among Lead SNPs using empirical prioritization based on SNP pairwise comparison. A. Input: 2,358 Lead SNPs associated with 467 diseases in NHGRI GWAS catalog and associated with 6301 mRNA expression levels in B Lymphoblastic cells by eQTL studies were considered, in pairwise, to find common biological mechanisms. **B. Hypothesis:** Surveyed Lead SNP pairs were then dichotomized into same disease and distinct disease Lead SNP pairs based on NHGRI GWAS catalog and into inter-inter, inter-intra and intra-intra pairs based on dbSNP genomic annotations. **C. Mechanism analytics:** These Lead SNP pairs (all three types) were prioritized by shared biological mechanisms via mRNA overlap derived from eQTL associations and similarity of biological process and/or molecular function based on gene ontology (GO) annotations of SNP-associated mRNAs. The prioritization was controlled by empirical resampling of SNP-mRNA associations and adjusted for multiple comparisons, in this case with an FDR<5% cutoff. **D. Validation of prioritization:** Prioritized SNP pairs were assessed for common regulatory properties derived from ENCODE data and were further validated in disease case studies. Note that we focused on SNP pairs with at least one intergenic SNP.

Supplementary Figure 2

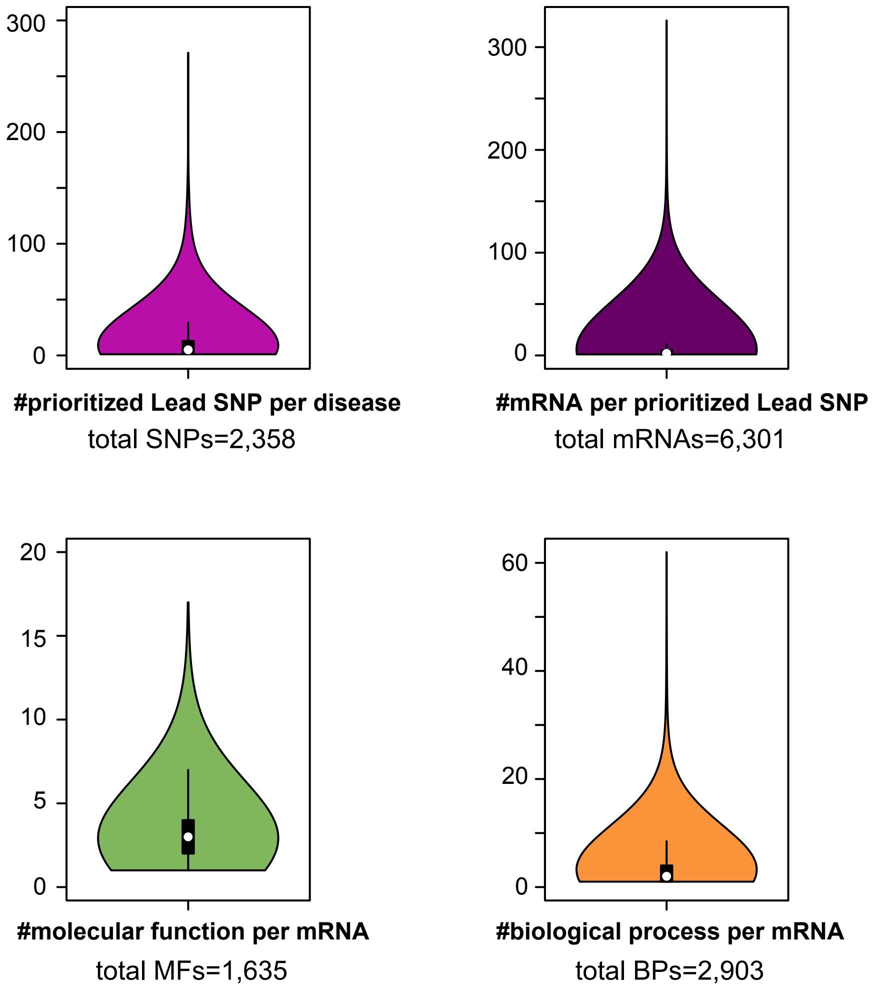

Supplementary Figure 2. Violin plot data distribution for each biological entity derived from input data. Input data includes 467 diseases, 2,358 Lead SNPs, 6,301 mRNAs, 1,635 molecular function annotations and 2,903 biological process annotations. On average (measured by median), each disease is associated with 5 SNPs; each SNP is associated with 2 mRNA transcripts by eQTL; and each mRNA is annotated with 3 molecular functions and 2 biological processes in the Gene Ontology knowledge base.

Supplementary Figure 3

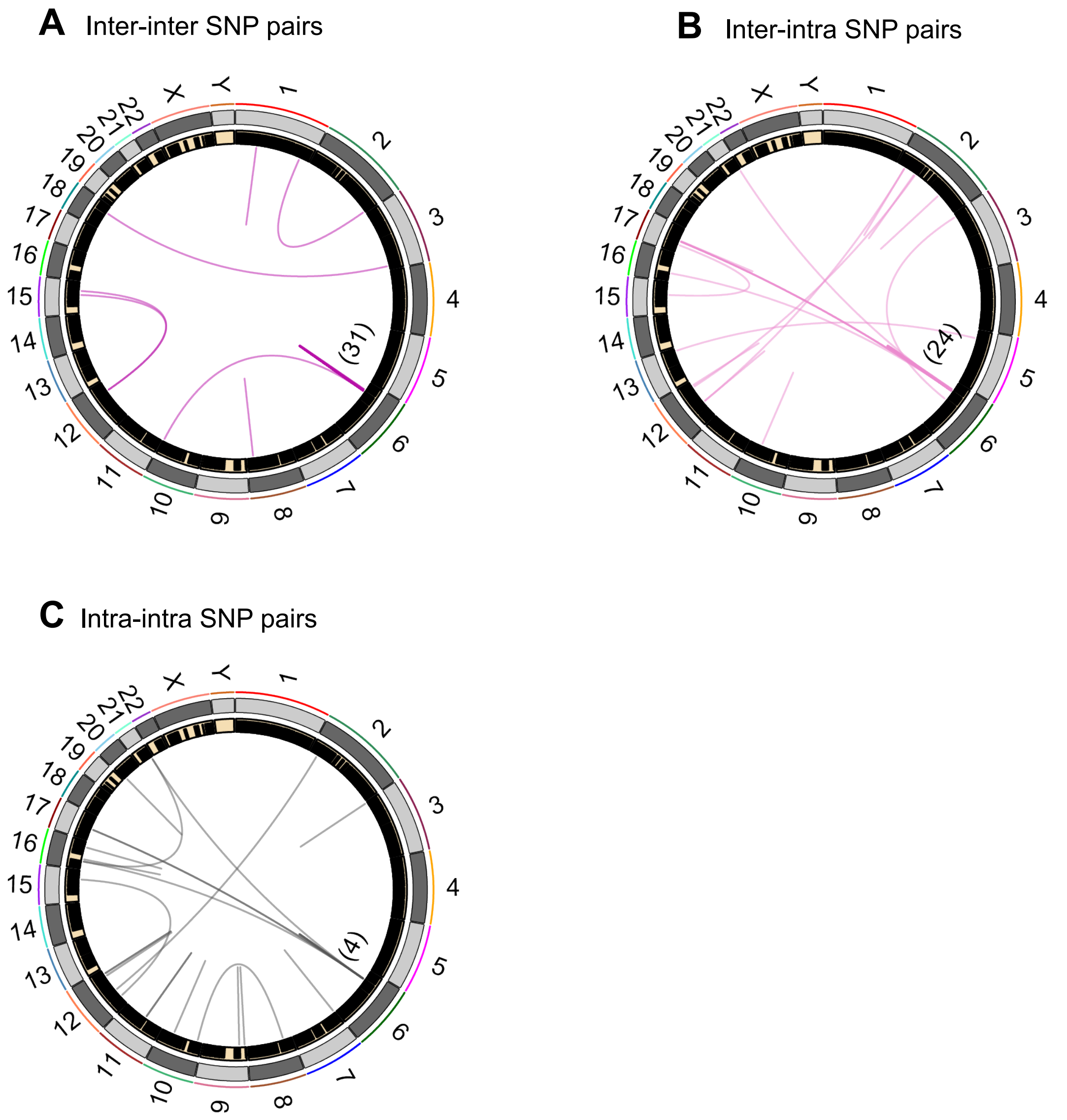

Supplementary Figure 3. Circos plot of SNP pairs prioritized within the same disease. Using the RCircos package (Zhang et al. BMC Bioinformatics 2013, 14:244), Lead SNP pairs associated within the same disease are shown: 38 inter-inter pairs (**Panel A**), 42 inter-intra pairs (**Panel B**), and 25 intra-intra pairs (**Panel C**). Multi-colored lines and alternating grey shaded blocks along the outer ring are used to represent chromosomes. SNPs are plotted according to chromosome and position with Lead SNP pairs joined by connecting lines. The inner ring shows syntenic blocks calculated across ten species using data from Larkin et al., Genome Research 2009, 19:770-777. Numbers in parentheses have been added next to connecting lines that map multiple Lead SNP pairs in close proximity at this resolution. Refer to **Supplementary Figure 4** for linkage and positional distances for within-chromosome SNP pairs.

Supplementary Figure 4

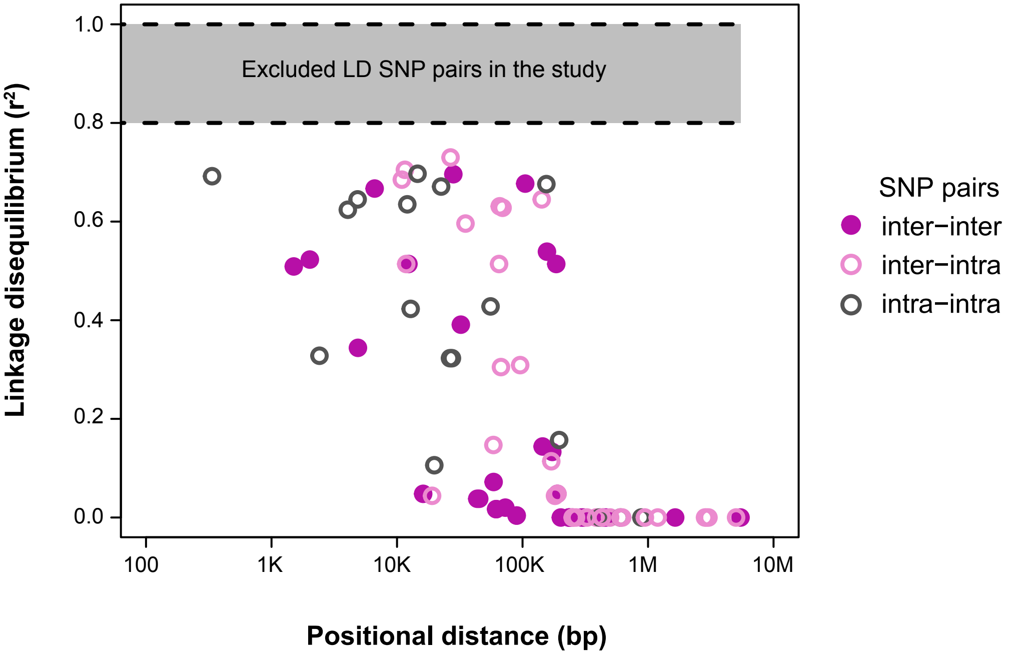

Supplementary Figure 4. Linkage and positional distance distribution of prioritized SNP pairs associated with the same diseases and mapped to the same chromosome. 81 within-disease Lead SNP pairs were identified as having both SNPs on the same chromosome. Log scale of positional distance (dbSNP build 138) is plotted on the x-axis with linkage disequilibrium (HapMap Phase III CEU; 4/19/2009) plotted on the y-axis for inter-inter (**filled purple circles**), inter-intra (**open pink circles**), and intra-intra (**grey**) Lead SNP pairs. The majority of pairs were more than 100,000 bp apart with very low LD ($r^2<0.01$), indicative of independence. SNP pairs with $r^2>0.8$ were excluded from analysis early in the process and therefore not part of these data, nor are SNP pairs where each mapped to a different chromosome (see **Supplementary Figure 3**).

Supplementary Figure 5

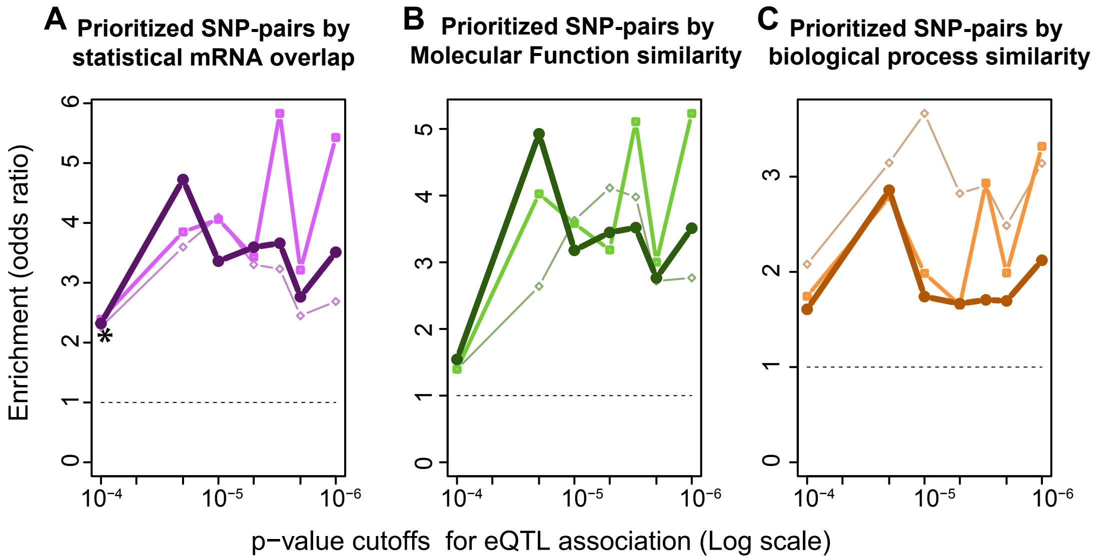

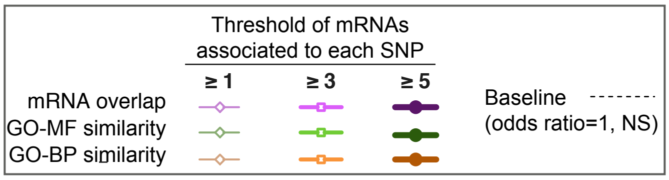

D Contingency table: *Exmaple panel A, Odds ratio 2.3

	Same disease SNP-pairs	Distinct disease SNP-pairs
Prioritized SNP-pairs	25	1,281
Non Prioritized SNP-pairs	11,812	1,369,265

SNP-pairs = intergenic-intragenic SNP-pairs

Supplementary Figure 5. Enrichment of shared biological mechanisms among <u>intergenic-intragenic Lead SNP pairs</u> associated with the same disease. Three biological mechanisms were imputed for each SNP pair with LD r^2<0.8: mRNA overlap **(A)**, the similarity of molecular functions **(B)**, and biological processes **(C)**. These Lead SNP pairs were prioritized and controlled empirically (100,000 permutation resampling; FDR<0.05). Prioritized intergenic-intragenic Lead SNP pairs were found significantly enriched in the same disease than across distinct diseases (odds ratio in y-axis) under various p-value cutoffs for eQTL associations (SNP-mRNA) (P-value in x-axis). A one-tailed Fisher's Exact Test (FET) was used to measure the significance of the enrichment. The odds ratios range from 2.3 to 5.8 ($3.6 \times 10^{-5} \leq p \leq 0.04$ for OR ≥ 3.5), 1.4 to 5.2 ($4 \times 10^{-4} \leq p \leq 0.04$ for OR ≥ 3.5), and 1.6 to 3.7 (best $p=8.1 \times 10^{-7}$) for intergenic-intragenic SNP pairs prioritized by mRNA overlap **(A)**, molecular function similarity **(B)**, and biological process similarity **(C)**, respectively. **(D)** The panel shows an example of the contingency table enrichment calculations for the cutoff of 10^{-4} in **Panel A** (see "*"). These results demonstrate that enrichment of common biological mechanisms is recapitulated among intergenic and intragenic Lead SNPs associated with the same disease beyond pairs that are exclusively intergenic SNPs.

Supplemental Figure 6

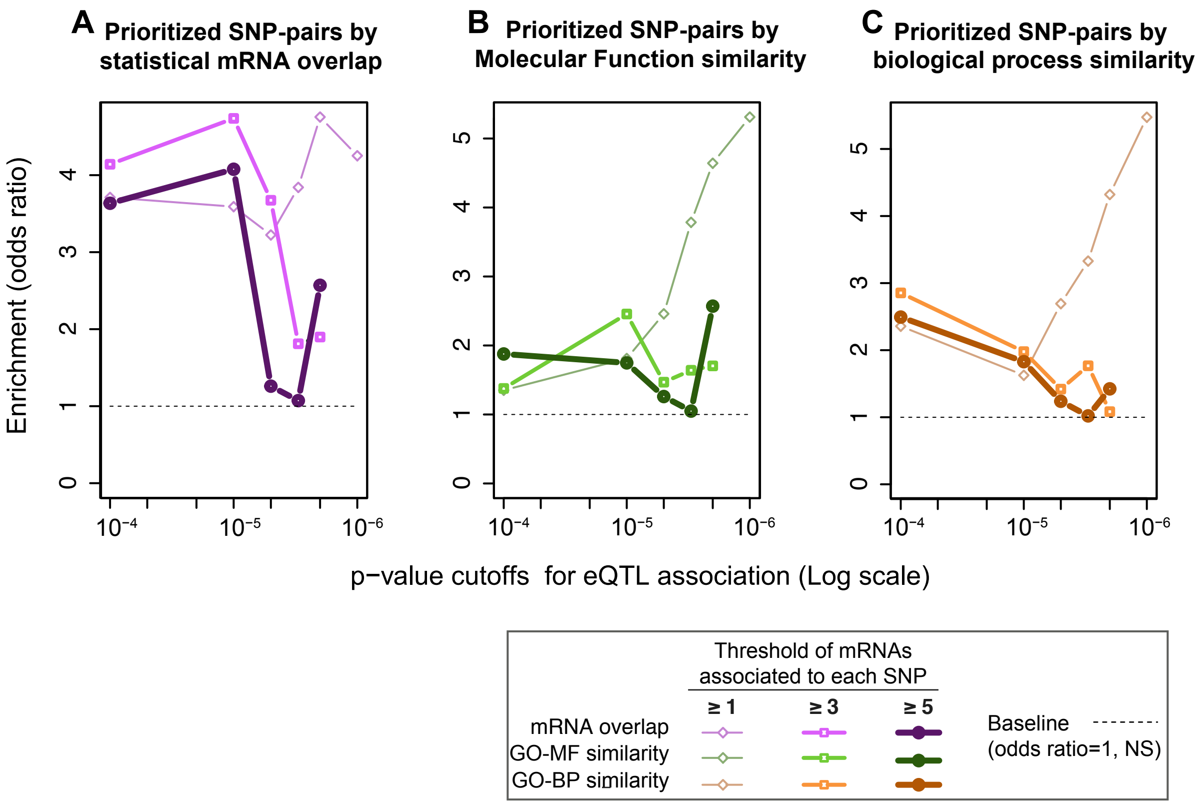

Supplementary Figure 6. Enrichment of shared biological mechanisms among <u>Intragenic-Intragenic Lead SNP pairs</u> associated with the same disease. Three biological mechanisms were imputed for each SNP pair with LD r^2<0.8: mRNA overlap **(A)**, the similarity of molecular functions **(B)**, and biological processes **(C)**. These Lead SNP pairs were prioritized and controlled empirically (100,000 permutation resampling; FDR<0.05). Prioritized intragenic-intragenic Lead SNP pairs were found significantly enriched in the same disease than across distinct diseases (odds ratio in y-axis) under various p-value cutoffs for eQTL associations (SNP-mRNA) (P-value in x-axis). A one-tailed Fisher's Exact Test (FET) was used to measure the significance of the enrichment.

Supplementary Figure 7

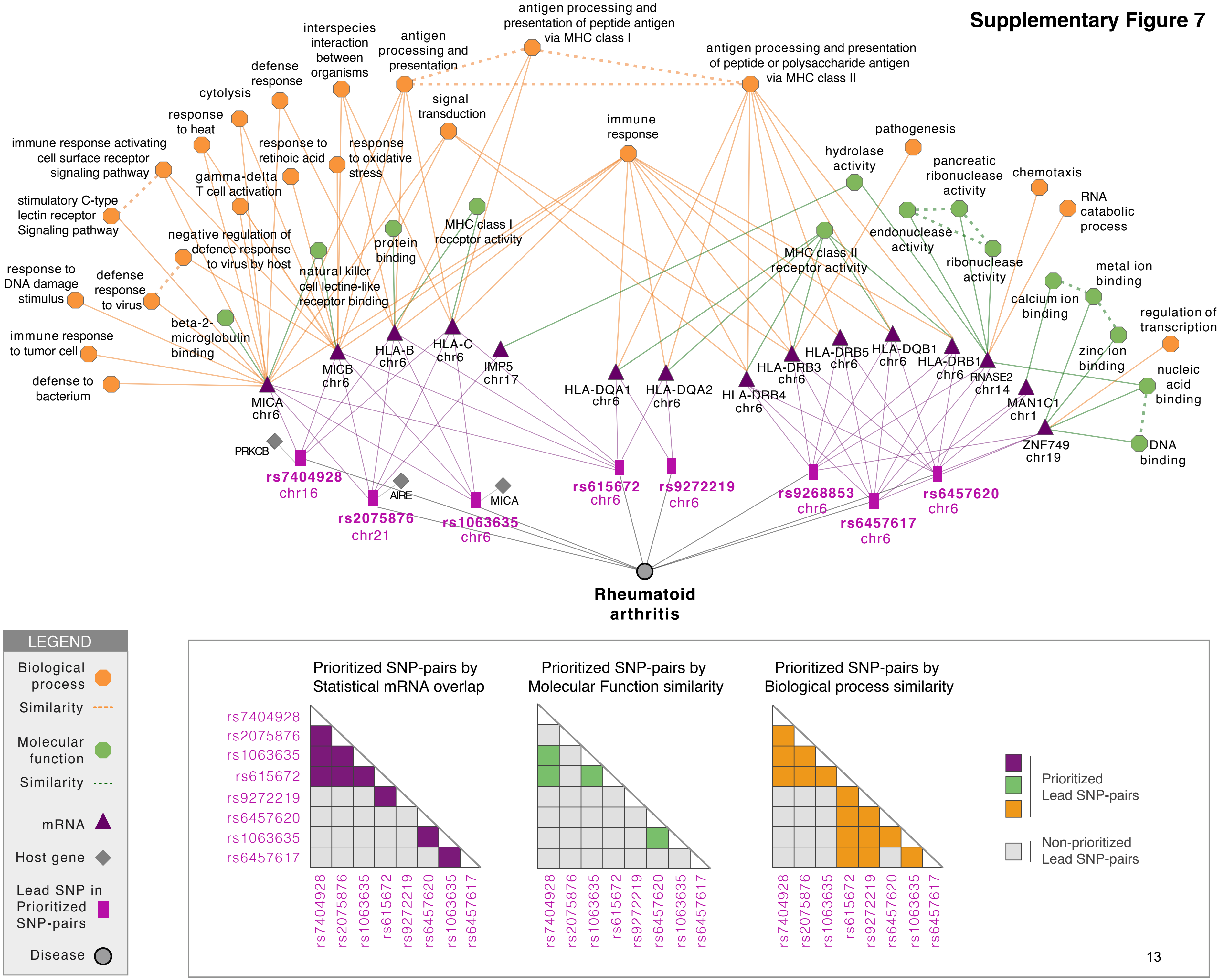

Supplementary Figure 7. A network of mRNA overlap and associated GO MF overlap and GO BP overlap of Lead SNP pairs associated with Rheumatoid arthritis. Those prioritized at FDR<5% are highlighted. This network translates the mechanistic map at a single disease level to reflect relationships between different biological scales and across Lead SNPs: from prioritized Lead SNP pairs and their eQTL-associated mRNAs to their associated disease-mechanisms (GO-MF and GO-BP). The network was visualized using Cytoscape (**Methods and Supplemental Methods**). The pairwise matrix (bottom) indicates each surveyed SNP pairs among those that were found prioritized at FDR<5% by mRNA overlap (purple square), by molecular function similarity (green square), and by biological similarity (orange square). The non-prioritized Lead SNP pairs are indicated by a grey square. The similarity between GO BP terms that share many genes and are hierarchically related is indicated by dotted lines. Computation of similarity is conducted by information theoretic distance (**Methods**).

Supplementary Figure 8

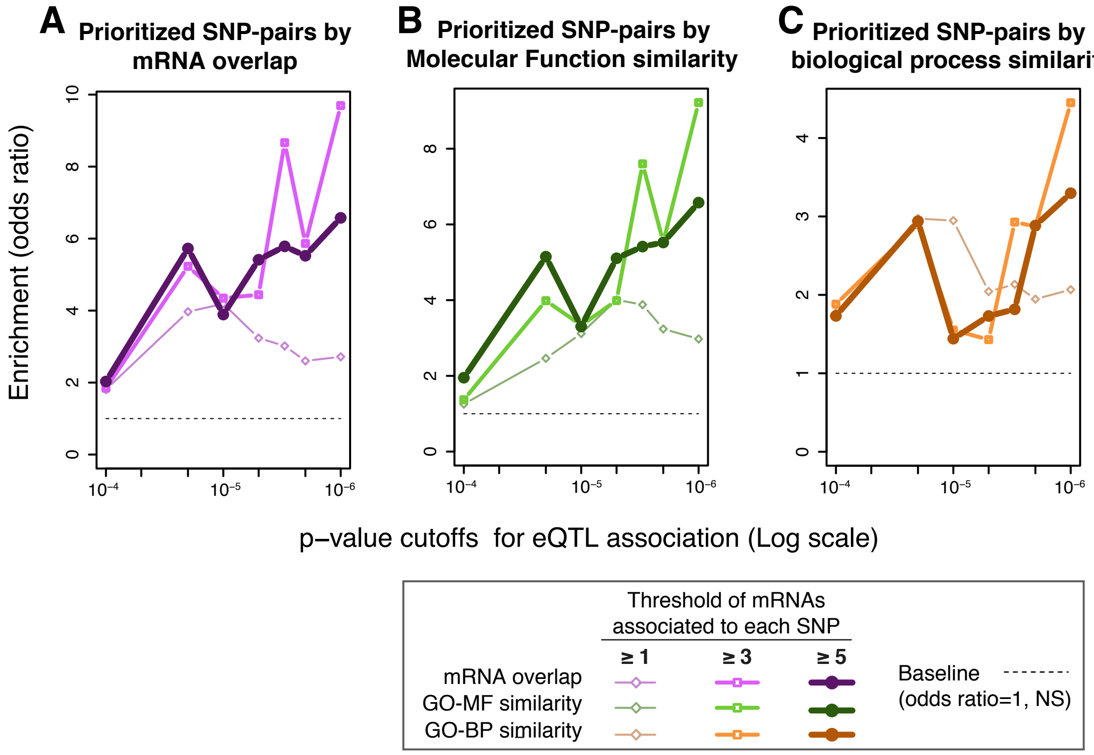

Supplementary Figure 8. Enrichment of shared biological mechanisms among inter-inter and inter-intra Lead SNP pairs with stringent <u>linkage disequilibrium cutoff (LD r^2 <0.01)</u> associated with the same disease. Three biological mechanisms were imputed for each SNP pair with stringent LD control (r^2<0.01): mRNA overlap **(A)**, the similarity of molecular functions **(B)**, and biological processes **(C)**. SNP pairs were prioritized and controlled empirically (100,000 permutation resampling; FDR<0.05). Prioritized inter-inter and inter-intra SNP pairs were found significantly enriched in the same disease than across distinct diseases (odds ratio in y-axis) under various p-value cutoffs for the eQTL association (SNP-mRNA) dataset (P-value in x-axis). A one-tailed Fisher's Exact Test (FET) was used to measure the significance of the enrichment. The odds ratios range from 1.8 to 9.7 ($1.3 \times 10^{-5} \leq p \leq 0.02$), 1.3 to 9.2 ($1.1 \times 10^{-4} \leq p \leq 0.02$ for OR ≥1.4), and 1.4 to 4.5 (best $p=1.4 \times 10^{-5}$) for inter-inter and inter-intra SNP pairs prioritized by mRNA overlap **(A)**, molecular function similarity **(B)**, and biological process similarity **(C)**, respectively. This demonstrated that enrichment of common biological mechanisms among Lead SNPs associated with the same disease (**Figure 3**) was an intrinsic property of the SNPs rather than the result of the linkage disequilibrium chosen.

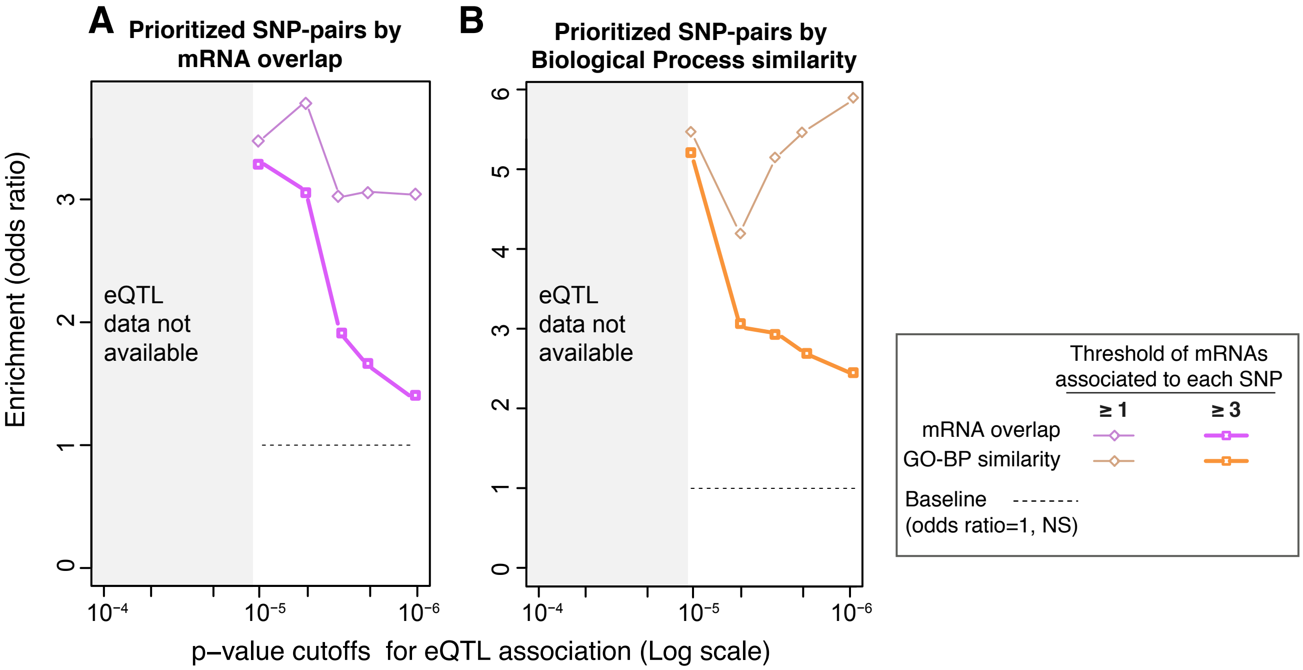

Supplementary Figure 9. Enrichment of shared biological mechanisms among inter-inter and inter-intra SNP pairs associated with the same disease using liver-derived eQTL associations. Two biological mechanisms were imputed for each SNP pair with LD r^2<0.8: mRNA overlap **(A)** and biological processes **(B)**. The two mechanisms were derived from liver eQTL data, only p≤10^{-5} associations were available (truncated part indicated in grey regions), and various cutoffs were shown in x-axis by a log scale. SNP pairs were prioritized and controlled empirically (100,000 permutation resampling; FDR<0.05). Prioritized inter-inter and inter-intra SNP pairs were found significantly enriched in the same disease than across distinct diseases (odds ratio in y-axis) under various p-value cutoffs for the eQTL association (SNP-mRNA) dataset (P-value in x-axis). A one-tailed Fisher's Exact Test (FET) was used to measure the significance of the enrichment. The odds ratios range from 1.4 to 3.8 ($6.1 \times 10^{-3} \leq p \leq 0.05$ for OR≥1.9) and 2.4 to 5.9 ($1.5 \times 10^{-12} \leq p \leq 0.04$ for OR≥2.7) for inter-inter and inter-intra SNP pairs prioritized by mRNA overlap **(A)** and biological process similarity **(B)**, respectively. These results demonstrated that the enrichment of common biological mechanisms among SNPs associated to the same disease (**Figure 3**) could be extended to other tissues or cell lines derived eQTL association dataset beyond LCL.

Supplementary Figure 10

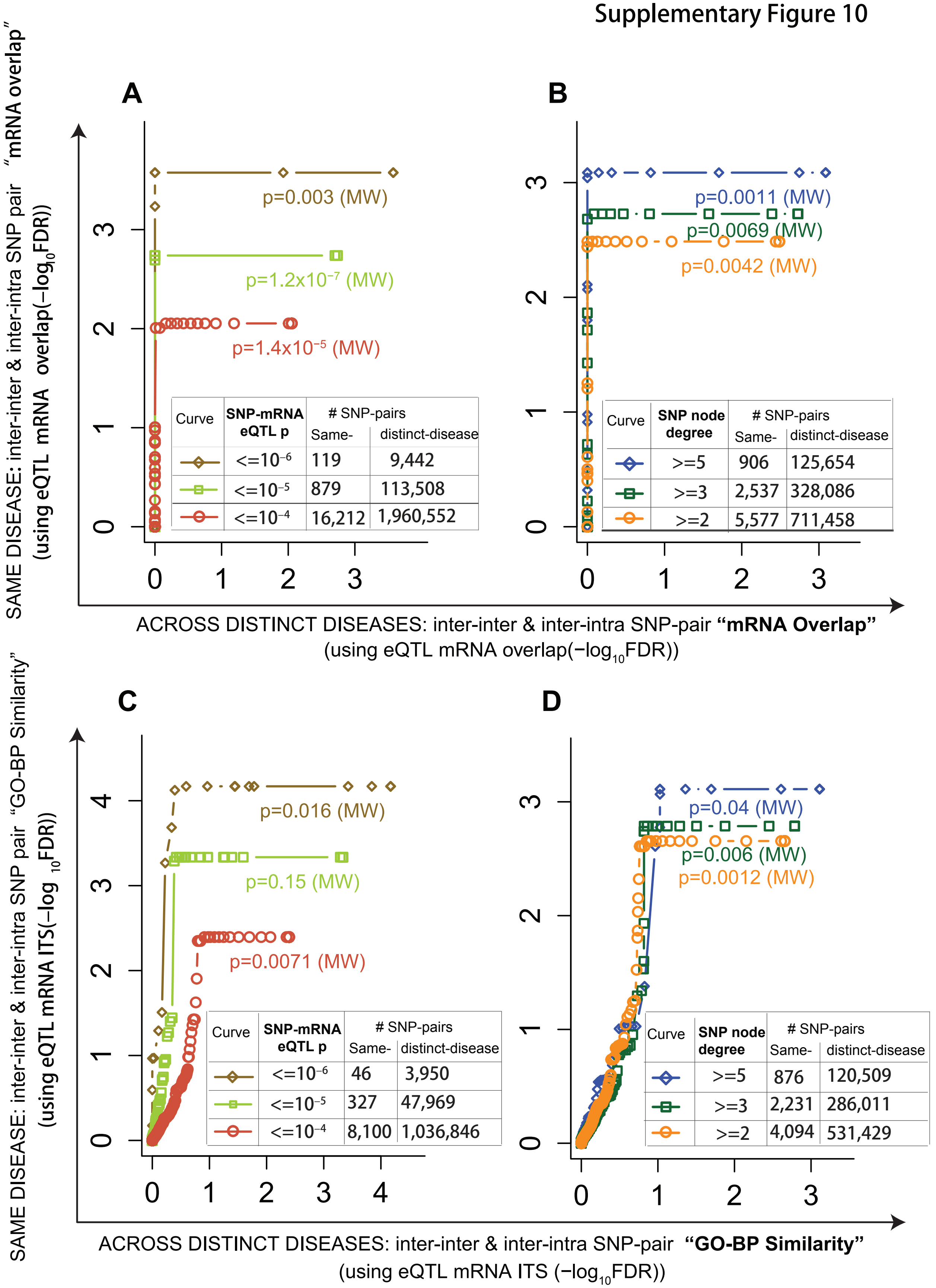

Supplementary Figure 10: Q-Q plots indicate that inter-inter and inter-intra SNP pairs associated with the same disease showed significantly different distributions of their shared mechanisms compared to those associated across distinct diseases. In all four panels, the left skewed Q-Q plots indicate that, in each quartile, the "same disease" distribution contains more significant pvalues than the "across distinct disease" distribution. **(A)** and **(B).** We generated Quantile-Quantile plots (Q-Q plots) to investigate two distributions of the significance of mRNA overlap between Lead SNP pairs: SNP pairs of the same disease vs. those of distinct diseases. **(C) and (D).** We generated the Q-Q plots to examine the distributions of Gene Ontology Biological Process similarity (GO-BP). In **(A)** and **(C)**, we calculated the Q-Q plots according to three different p-value cutoffs of eQTL associations (**Methods, Supplementary Methods**). In **(B)** and **(D)**, we calculated the Q-Q plots according to three different SNP node degree cutoffs (**Methods, Supplementary Methods**). Also, we calculated the Mann-Whitney U tests for each of the three curves in each panel (one-sided; shown alongside and color-coded according to each Q-Q plot curve). The significance of overlapping mRNAs and mRNA biological similarity of a Lead SNP pair was calculated empirically from 100,000 conservative permutations of the LCL eQTL associations and was adjusted by false discovery rate (overlap FDR or ITS FDR, shown in log scale in axis) (**Methods**). Of note, the horizontal plateau of p-values are attributable to data being truncated at $p=10^{-5}$ (related to the number of permutations). SNP pairs with linkage disequilibrium $r^2 \geq 0.01$ were excluded from this study. Other cutoffs for eQTL data led to similar results (data not shown).

Supplementary Figure 11

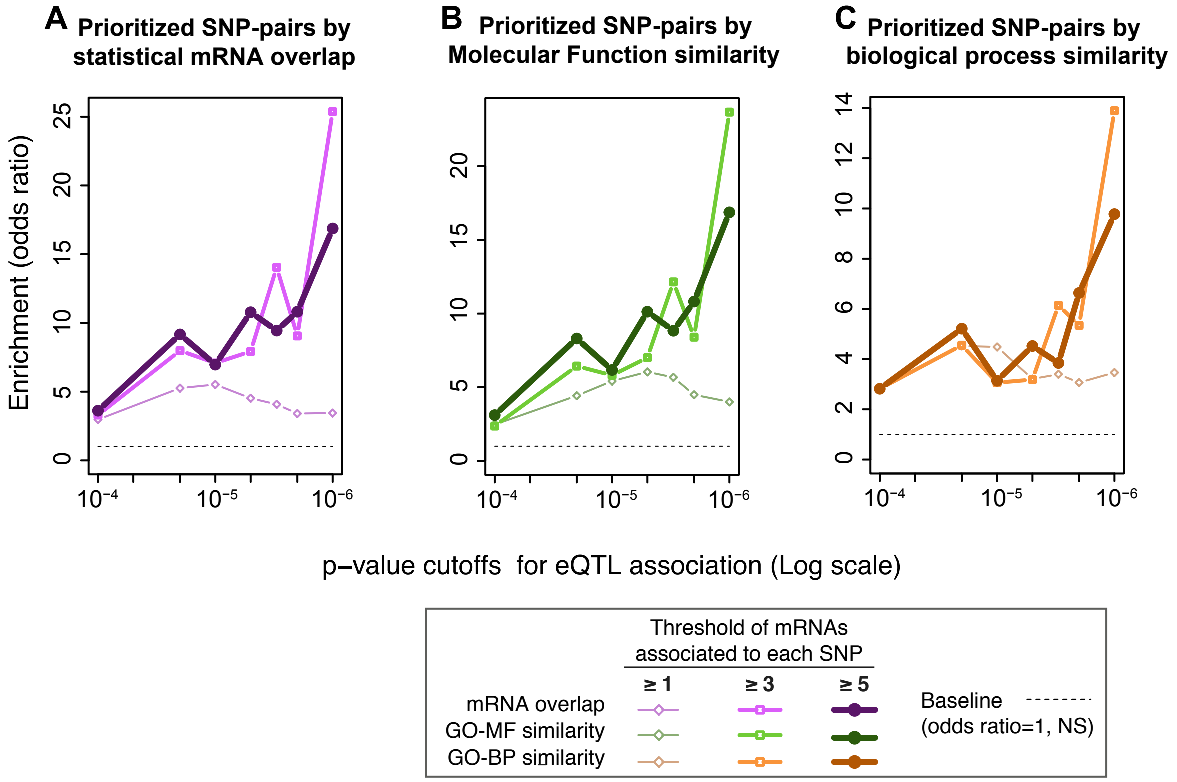

Supplementary Figure 11. Enrichment of shared biological mechanisms among inter-inter and inter-intra Lead SNP pairs associated within the same disease remains consistent following analysis using an <u>alternative genome annotation</u>. Intergenic and intragenic SNP assertions were calculated from dbSNP Build 138 and GENCODE version 19 (protein coding, miRNA, InRNA) with intergenioc SNPs defined as at least 2000bp 5' and 500bp 3' of both protein-coding and noncoding gene coordinates. Three biological mechanisms were imputed for each SNP pair: mRNA overlap **(A)**, the similarity of molecular functions **(B)**, and similarity of biological processes **(C)**. SNP pairs were prioritized and controlled empirically (100,000 permutation resampling; FDR<0.05). See text accompanying Figure 3 for complete details. Prioritized inter-inter and inter-intra Lead SNP pairs were significantly enriched for biomodule similarity for increasing levels of eQTL association (SNP-mRNA) stringency. Enrichment was calculated "within disease" versus across "distinct diseases" using a one-tailed Fisher's Exact Test (FET). Comparing with that in Figure 3, higher odds ratio were obtained. The odds ratios range from 3.0 to 25.4 ($1.1x10^{-12} \leq p \leq 1.6x10^{-4}$), 2.4 to 23.7 ($1.7x10^{-11} \leq p \leq 3.2x10^{-4}$), and 2.8 to 13.9 ($1.8x10^{-15} \leq p \leq 5.9x10^{-3}$) for inter-inter and inter-intra SNP pairs prioritized by mRNA overlap **(A)**, molecular function similarity **(B)**, and biological process similarity **(C)**, respectively. This demonstrated that enrichment of common biological mechanisms among Lead SNPs associated with the same disease (**Figure 3)** was an intrinsic property of the SNPs rather than the choice of a specific human reference genome annotation and gene definition.

Supplementary Figure 12

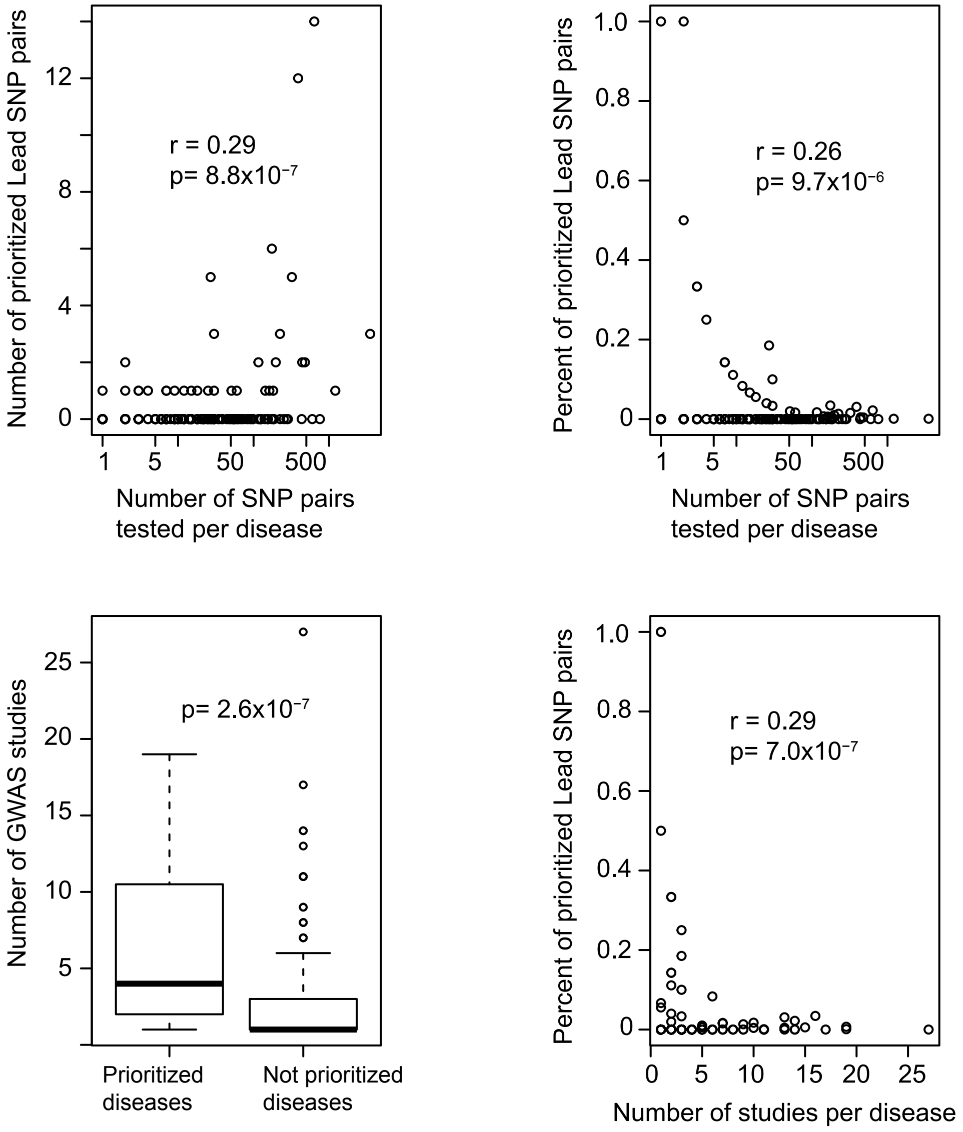

Supplementary Figure 12. GWAS input covariates contributing to the interpretation of study results. The number of prioritized Lead SNP pairs within a disease is modestly correlated with the total number of SNPs associated by GWAS to a disease **(A)**. Similarly, the proportion (percent) of prioritized Lead SNPs associated by GWAS to a disease is also slightly correlated with the total number of SNPs associated by GWAS to a disease – though this may be more complex (bimodal distribution) as the plot shows a smaller subset of anticorrelated patterns **(B)**. Diseases overrepresented in GWAS studies are also overrepresented in our results **(C)**. Percent of prioritized Lead SNP pairs within a disease increases imperceptibly with the number of GWAS studies for that disease **(D)**. Correlations were assessed by a non-parametric test (Spearman; A, B, D) and difference by Mann-Whitney U test (C).

Supplementary Figure 13

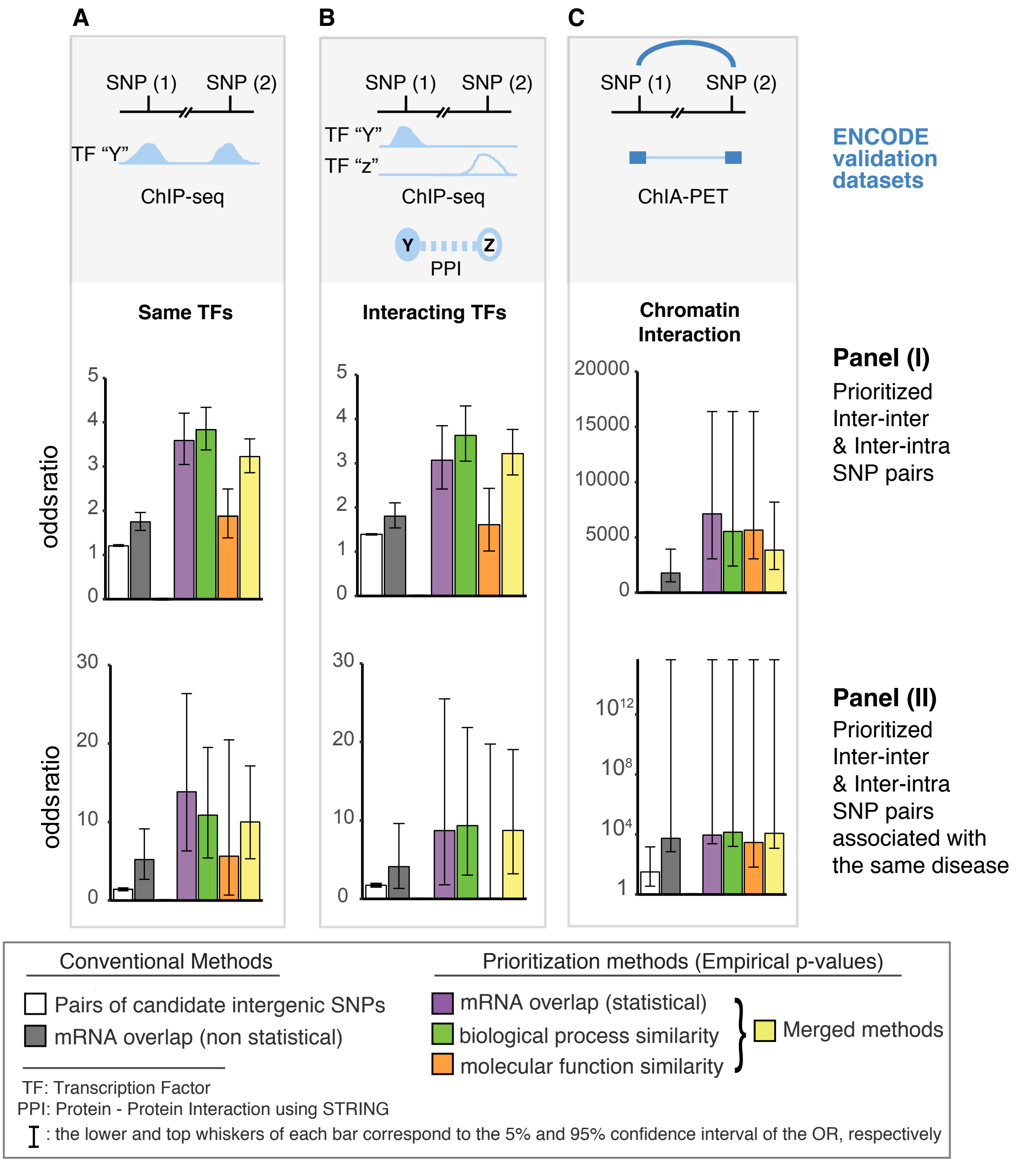

Supplementary Figure 13. Prioritized inter-inter and inter-intra Lead SNP pairs with linkage disequilibrium $\underline{r^2 \leq 0.01}$ are also enriched in genomic regions sharing common ENCODE-derived transcription factors (TFs) and regulatory elements. This figure examines the reproducibility of results in Figure 5 using a more stringent LD cutoff of $r^2<0.01$. ENCODE data were used to assess the propensity of prioritized inter-inter and inter-intra Lead SNP pairs to localize in regulatory regions with **(A)** the same TF(s) via ChIP-seq, **(B)** two distinct interacting TFs (ChIP-seq and protein-protein interactions, PPI), and **(C)** long-range chromatin interaction properties (ChIA-PET). Enrichment of inter-inter and inter-intra Lead SNP pairs (odds ratios with 95% confidence intervals, y-axis) in regions sharing common regulatory properties were evaluated between (i) prioritized and non-prioritized Lead SNP pairs (**Panel I**), (ii) prioritized Lead SNP pairs in the same disease and across-diseases (**Panel II**). Greater ORs are observed in disease-specific SNP pairs (Panel II compared to Panel I); ORs range from 1.2 to 7129.2 ($2x10^{-16} \leq p \leq 0.02$) in **Panel I** and 1.4 to 14140.2 ($1.6x10^{-31} \leq p \leq 0.05$; one exception) in Panel II. The odds ratios are comparable to those yielded by inter-inter and inter-intra SNP pairs of LD $r^2<0.08$. Candidate inter-inter and inter-intra SNPs considered for the enrichments were associated with mRNAs by eQTL with $p \leq 10^{-4}$ (mRNA overlap; grey bars). Stringent prioritizations using empirical computations were performed on mRNA overlap (mauve bars), biological process similarity (green bars), molecular function similarity (orange bars), and in combination (merged methods; yellow bars). Enrichments of SNP pairs were performed using Fisher's exact test among all pairwise combinations of NHGRI disease-associated SNPs. Potential causal SNPs represented by the Lead SNPs in the pairs were included in this regulatory function study and were taken from RegulomeDB (**Materials and Methods**).

Supplemental Table 1. Description of prioritized Lead SNP-pairs using mRNA associations to SNPs. Depending on the cutoff, ~ 71%-77% of prioritized Lead SNP-pairs are intergenic. Each Lead SNP-pair may be prioritized by more than one mechanism, and therefore the total count of mechanisms per column may exceed 100%. Inter-inter and inter-intra Lead SNP-pairs comprise of at least one intergenic Lead SNP, whereas intra-intra Lead SNP-pairs comprise exclusively of intragenic SNPs. Only Lead SNP-pairs with linkage disequilibrium LD, r2 <0.8, were considered in the study.

		INPUT	OUTPUT of Prioritized Lead SNP-pairs				
			p<5%	p<1%	FDR 50%	FDR 25%	FDR 5%
Lead SNP-pairs (#)	Inter-inter & inter-intra SNP-pairs	1,977,927	109,954	32,202	15,205	8,302	3,870
	Intra-intra SNP-pairs	800,438	43,443	11,908	5,172	2,591	1,141
	Total	2,778,365	153,397	44,110	20,377	10,893	5,011
Lead SNP-pairs (#) prioritized by distinct mechanism*	by Statistical mRNA Overlap	n/a	7,441	6,111	3,791	3,289	2,649
	by Molecular Function (GO-MF)	n/a	83,883	21,944	5,360	2,821	1,412
	by Biological Processes (GO-BP)	n/a	77,281	22,400	15,381	8,295	3,856

* Distinct mechanisms are imputed from mRNAs in eQTL associations to Lead SNPs at p<10-4 **(Methods)**

Supplementary Table 2. Detailed description of the datasets and databases used in the study.

A- Association and knowledge-based Datasets

Name	Description	Source	Download date	Refer to
eQTL associations	**SNP-mRNA** associations in Lymphoblastoid cell lines, LCLs	www.scandb.org	10-11-10	All Figures
	**SNP-mRNA** associations in liver	www.scandb.org	08-15-13	Supp. Fig. S9
NHGRI GWAS catalog	**SNP-disease/trait** associations	www.genome.gov/gwastudies/	06-07-12	All Figures & Supp.
dbSNP 138	**Intergenic and intragenic SNP definitions of the main study**	ftp://ftp.ncbi.nlm.nih.gov/snp/organisms/human_9606/ASN1_flat/	02-21-14	All Figures & Supp.
GENCODE 19	**Intergenic and intragenic SNP alternate definitions**	http://www.gencodegenes.org/releases/	03-24-14	Supp. Fig. 11
HapMap (Caucasian population)	**SNP-pairs**: Linkage Disequilibrium (LD) $r^2 < 0.8$	http://hapmap.ncbi.nlm.nih.gov/	04-19-09	All Figures & Supp.
RegulomeDB	**Lead SNPs - LD SNPs**: Linkage Disequilibrium (LD) $r^2 \geq 0.8$	http://regulomedb.org/GWAS/index.html	07-06-12	Fig. 5<br>Supp. Fig. 13
Gene Ontology (GO)	**Gene - GO terms**<br>GO-BP: Biological Process<br>GO-MF: Molecular Functional	(ftp://ftp.ncbi.nlm.nih.gov/gene/DATA/gene2go.gz	19-05-09	All Figures & Supp.
STRING v9.1	**Protein-Protein interactions**	http://string-db.org/	08-22-11	Fig. 5 & Supp. Fig. 13

B- dbGaP dataset for genetic risk interaction studies

Dataset Name	Study Accession	Source	Cohort	Refer to
CGEMS BladderCancer_	phs000346.v1.p1	http://www.ncbi.nlm.nih.gov/gap/?term=phs000346.v1.p1	8646	Table 1
GenADA/LONG/Imaging (Alzeimer's Disease)	phs000346.v1.p1	http://www.ncbi.nlm.nih.gov/gap/?term=phs000219.v1.p1	733	Table 1

C- ENCODE Datasets

Assay	Cell type	Description	Source	Download date	Refer to
TfbsClustered	Multiple	Multiple TFs	wgEncodeRegTfbsClusteredWithCellsV3.bed.gz	05-25-13	Fig. 5 & Supp. Fig. 13

Supplementary Table 2 continued.

ChIA-PET	MCF-7	CTCF	http://chiapet.gis.a-star.edu.sg/downloads/encode-datasets	09-05-13	Fig. 5 & Supp. Fig. 13
	MCF-7	RNAPII (Pol II)		09-05-13	Fig. 5 & Supp. Fig. 13
	MCF-7	ER		09-05-13	Fig. 5 & Supp. Fig. 13
	K562	RNAPII (Pol II)		09-05-13	Fig. 5 & Supp. Fig. 13
	HeLa-S3	RNAPII (Pol II)		09-05-13	Fig. 5 & Supp. Fig. 13
	HCT-116	RNAPII (Pol II)		09-05-13	Fig. 5 & Supp. Fig. 13
	NB4	RNAPII (Pol II)		09-05-13	Fig. 5 & Supp. Fig. 13

Supplementary Table 3: **Characteristics of the Rheumatoid Arthritis cohort used for interaction testing.**

	Cases N=1,115	Controls N=24,169
Age (years)*	56.2±17.8	53.4±23.9
% female	74.3%	51.5%
Record length (years)*	11.7±5.1	8.5±5.7

*mean±SD

Supplementary Table 4. List of abbreviations and key concept definitions.

Abbreviation	Definition/Description
**ChIA-PET**	Chromatin Interaction Analysis by Paired-End Tag Sequencing. Method that combines ChIP-based methods and Chromosome Conformation Capture (3C) to assess interactions between any genomic regions containing a particular DNA binding protein
**ChIP-seq**	High-throughput method that combines chromatin immunoprecipitation (ChIP) and massively parallel sequencing, to survey interactions between protein, DNA, and RNA
**DNase-seq**	High resolution mapping of DNase I hypersensitive sites (HS) for identifying all different types of regulatory elements
**ENCODE**	ENCyclopedia of DNA Elements is a comprehensive annotation of functional elements in the human genome based on a variety of biochemical assays performed across multiple tissues and cell lines
**eQTLs**	Expression Quantitative Trait Loci. Correlation-based method to associate SNP to mRNA expression levels within a given populations
**FDR**	False Discovery Rate
**FET**	Fisher's Exact Test. Statistical significance test assessing the association between two properties/variables
**GO, GO-MF and GO-BP**	Gene ontology. Annotation of molecular function and biological process of genes and gene products
**GWAS**	Genome-wide association studies assess a correlation between SNPs and disease occurrence within a given population
**Intergenic SNP (inter)**	Intergenic SNPs are SNPs localized outside the intragenic regions (Intragenic = 2kb upstream the transcription starting site and 0.5kb downstream the termination site of a gene)
**Lead SNP-pairs**	Pairs of SNPs: (i) inter-inter (ii) inter-inter and (iii) intra-intra
**Intragenic SNP (intra)**	SNPs located in intragenic regions which boundaries extend 2kb upstream the transcription starting site and 0.5kb downstream the termination site of a gene
**ITS**	Information Theoretical Similarity
**LCL**	Lymphoblastoid Cell Line. Patient- or individual-derived peripheral B lymphocytes transformed and immortalized by Epstein-Barr virus (EBV)
**LD SNPs or proxies**	SNPs with a strong Linkage Disequilibrium (LD $r^2 \geq 0.8$) with Lead SNPs
**Lead SNPs**	GWAS disease-SNPs
**Lead SNPs in the prioritized SNP-pairs**	GWAS disease-SNPs found among prioritized SNPs sharing common biological mechanisms
**MDR**	Multifactor Dimensionality Reduction
**MF**	Molecular Function
**mRNA node degree**	Number of SNPs associated with each mRNA
**OR**	Odds Ratio. Statistical measure of the effect size; strength of association between two properties/variables
**PheWAS**	Phenome-Wide Association Study
**PPI**	Protein-Protein Interaction used to assess the level of interaction between transcription factors (TF)
**Prioritized SNP-pairs or Prioritized Lead SNP-pairs**	Lead SNP-pairs that yielded sufficient statistical significance by any of the prioritization methods: (i) mRNA overlap, (ii) GO-MF similarity, and (iii) GO-BP similarity
**Q-Q Plot**	Quantile-quantile plots
**Regulatory SNPs**	SNPs localized in regulatory elements often in intergenic regions
**SNP node degree**	Number of mRNAs associated by eQTL to each SNP
**SNP**	Single Nucleotide Polymorphism
**TF**	Transcription Factor
**TFBS**	Transcription Factor Binding Sites
